# FGF Signalling Regulates Chromatin Organisation during Neural Differentiation via Mechanisms that Can Be Uncoupled from Transcription

**DOI:** 10.1371/journal.pgen.1003614

**Published:** 2013-07-18

**Authors:** Nishal S. Patel, Muriel Rhinn, Claudia I. Semprich, Pamela A. Halley, Pascal Dollé, Wendy A. Bickmore, Kate G. Storey

**Affiliations:** 1 Division of Cell & Developmental Biology, College of Life Sciences, University of Dundee, Dundee, United Kingdom; 2 Institut de Génétique et de Biologie Moléculaire et Cellulaire, Centre National de la Recherche Scientifique (UMR 7104), Institut National de la Santé et de la Recherche Médicale (U 964), Université de Strasbourg, Illkirch-Strasbourg, France; 3 MRC Human Genetics Unit, MRC Institute of Genetics and Molecular Medicine, University of Edinburgh, United Kingdom; Hubrecht Institute, Netherlands

## Abstract

Changes in higher order chromatin organisation have been linked to transcriptional regulation; however, little is known about how such organisation alters during embryonic development or how it is regulated by extrinsic signals. Here we analyse changes in chromatin organisation as neural differentiation progresses, exploiting the clear spatial separation of the temporal events of differentiation along the elongating body axis of the mouse embryo. Combining fluorescence in situ hybridisation with super-resolution structured illumination microscopy, we show that chromatin around key differentiation gene loci *Pax6* and *Irx3* undergoes both decompaction and displacement towards the nuclear centre coincident with transcriptional onset. Conversely, down-regulation of *Fgf8* as neural differentiation commences correlates with a more peripheral nuclear position of this locus. During normal neural differentiation, fibroblast growth factor (FGF) signalling is repressed by retinoic acid, and this vitamin A derivative is further required for transcription of neural genes. We show here that exposure to retinoic acid or inhibition of FGF signalling promotes precocious decompaction and central nuclear positioning of differentiation gene loci. Using the *Raldh2* mutant as a model for retinoid deficiency, we further find that such changes in higher order chromatin organisation are dependent on retinoid signalling. In this retinoid deficient condition, FGF signalling persists ectopically in the elongating body, and importantly, we find that inhibiting FGF receptor (FGFR) signalling in *Raldh2−/−* embryos does not rescue differentiation gene transcription, but does elicit both chromatin decompaction and nuclear position change. These findings demonstrate that regulation of higher order chromatin organisation during differentiation in the embryo can be uncoupled from the machinery that promotes transcription and, for the first time, identify FGF as an extrinsic signal that can direct chromatin compaction and nuclear organisation of gene loci.

## Author Summary

Changes in the position of genes within the nucleus and in their local organisation frequently correlate with whether or not genes are turned on. However, little is known about how such nuclear organisation is controlled and whether this can be separated from the mechanisms that promote transcription. We show here that central nuclear position and chromatin de-compaction correlate with onset of expression at key neural differentiation gene loci in the mouse embryo. Conversely, the locus of a gene that is down-regulated as neural differentiation commences exhibits a shift towards the nuclear periphery as this takes place. Importantly, we show that signalling through the fibroblast growth factor (FGF) pathway regulates changes at this level of nuclear organisation. FGF represses differentiation gene transcription and keeps differentiation gene loci compact and at the nuclear periphery. By blocking FGF signalling in a retinoid deficient embryo in which differentiation genes are not expressed, we further show that control of nuclear organisation by FGF is not just a consequence of gene transcription. These findings are the first to demonstrate that such higher order nuclear organisation is regulated in the developing embryo, that this takes place downstream of FGF signaling, and can be uncoupled from the machinery of gene transcription.

## Introduction

Differentiation is directed by extrinsic signals that regulate expression of transcription factors, which determine cell fates. A further critical level of regulation is provided by so-called higher order chromatin organisation, which includes changes in local chromatin compaction and nuclear position of gene loci. Such changes have been documented in *in vitro* differentiation assays, but this level of organisation has not been analysed as extensively during embryonic development and the role of signalling pathways in modulating chromatin and nuclear organisation in the developing embryo remains unexplored.

**Figure 1:**
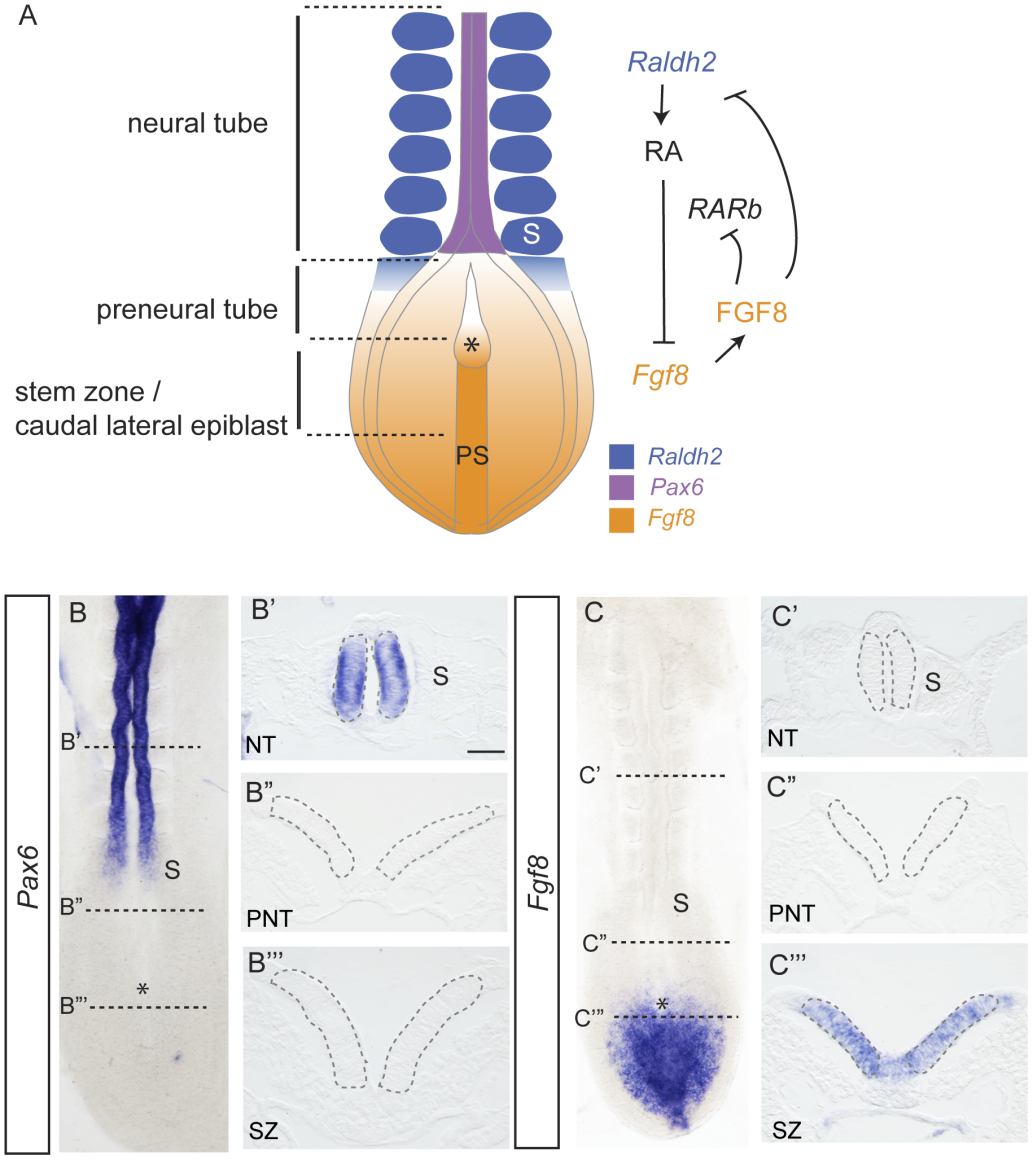
Signals regulating differentiation and expression patterns of *Pax6* and *Fgf8* along the elongating neural axis. (A) Summary of cell populations, signal localisation and interactions at the caudal end of the E8-8.5 mouse embryo, RA retinoic acid, RAR, retinoic acid receptor, FGF, fibroblast growth factor, PS, primitive streak, S, somite, *Raldh2, Retinaldehyde dehydrogenase 2*; (B) *Pax6* is expressed in the neural tube (B′) in transverse section (TS), but not in (B″) preneural tube or (B‴) stem zone; (C) *Fgf8* is expressed in the stem zone, but not in the neural tube (C′) in TS or in (C″) preneural tube, stem zone expression in TS (C‴). Grey dashed lines in B′, B″, B‴, C′,C″, C‴ outline cell populations in which nuclei were assessed in FISH experiments. Scale bar = 50 microns, asterisk indicates position of the node in all embryo images.

During vertebrate embryonic development, induction of the future brain is followed by the progressive generation of neural tissue as the body axis elongates and this provides a unique opportunity to investigate steps leading to the onset of neural differentiation. New neural tissue arises from the stem zone/caudal lateral epiblast (adjacent to the primitive streak), which includes resident axial stem cells [1], [2] (Figure 1A). As cells leave this regressing region they either ingress to form paraxial mesoderm or remain in the epiblast and commence neural differentiation. Stem zone cells are highly proliferative and are maintained by FGF and Wnt signalling [3], [4]. This is attenuated by retinoid signals synthesised in the forming somites [3], [5], [6] (Figure 1A). Retinoic acid (RA) promotes neural differentiation in at least two steps; first repressing FGF/Wnt signalling and then promoting expression, in the forming neural tube, of key genes characteristic of neural progenitors, such as *Sox1, Sox3* and *Pax6* [3], [7]–[9]. Importantly, FGF signalling also counteracts retinoid signalling, repressing expression of *Raldh2* which encodes retinaldehyde dehydrogenase 2 - an RA synthesising enzyme - in the presomitic mesoderm and RA receptor beta (*RARb*) in the forming neuroepithelium as well as promoting expression of *Cyp26a1*, encoding a RA catabolising enzyme reviewed in [1] (summarised in Figure 1A).

The underlying molecular mechanisms through which this opposing signalling switch controls differentiation onset in the embryonic body axis are not well understood, but might involve changes in gene expression determined by altered chromatin structure around target gene loci. One way in which chromatin compaction is locally regulated is by the action of polycomb repression complexes (PRC); and there is some evidence implicating FGF signalling in the regulation of polycomb complex component genes. Polycomb group (PcG) proteins are key regulators of cell growth and differentiation genes [10]–[12] and are found in two broad classes of complex; PRC2, which mediates the histone modification H3K27me3 associated with transcriptional repression through the activity of the Ezh1/2 histone methyltransferase and PRC1, which mediates local chromatin compaction [13]. In the zebrafish, the epiboly/tailbud phenotype of Ph2α morphants (homologue of the PRC1 component polyhomeotic) is similar to that of *Fgf8* morphants, and Ph2α acts downstream of FGF signalling, which is necessary, although not sufficient for Ph2α expression [14]. Mice mutant for *Fgf8* or for PcG genes (*Eed, Ezh2 or Ring1b*) also share a common gastrulation failure phenotype, with some reported proliferation defects [15]–[19], suggesting conservation of a relationship between FGF signalling and polycomb function in the early embryo.

Retinoic acid can signal directly to chromatin via liganded retinoic acid receptor – retinoid X receptor (RAR-RXRs) heterodimers and their sequence specific binding to retinoic acid response elements (RAREs) and this is known to attenuate binding of PRC2 components and to decrease H3K27me3 enrichment at these sites [20]–[22]. These observations suggest that in some contexts FGF may promote, while retinoid signalling represses, the action of polycomb complexes. Furthermore, as activation of polycomb target loci, such as the Hox gene clusters, is accompanied by visible unfolding of the compact state [23], [24], such signals might alter chromatin compaction at differentiation gene loci. Importantly, changes in chromatin compaction and local organisation are not simply a consequence of transcription; experimental translocation of a *3′ Hoxb1* transgene to the 5′ end of the Hoxd cluster elicited such chromatin changes in a cellular context in which *Hoxb1* is not transcribed [25]. This phenomenon shows that alteration of chromatin organisation can prefigure gene transcription.

A further important manifestation of higher order chromatin organisation that frequently correlates with transcription is the position of a locus with respect to the nuclear periphery, which can be a repressive environment. Although recent studies have shown that artificial tethering to the nuclear periphery need not necessarily lead to gene silencing [26]–[28], many loci do exhibit a change in distance to the nuclear edge, and association with the nuclear lamina [29] which correlates with their potential for transcription. As extrinsic signals orchestrate development by directing gene transcription, it is likely that this involves regulation of such higher order organisation, however, it is not known whether particular signalling pathways direct such mechanisms nor whether they can elicit changes in chromatin organisation independently of transcriptional regulation.

To assess changes in higher-order chromatin organisation during the progressive generation of neural tissue in the elongating body axis of the mouse embryo, we used fluorescence in situ hybridisation (FISH) combined with super-resolution structured illumination microscopy (SIM). We analysed changes in higher-order chromatin organisation at the loci of exemplar neural progenitor genes *Pax6* and *Irx3* as differentiation takes place and compared this with the *Fgf8* locus, which is transcriptionally downregulated as cells leave the stem zone (Figures 1B–B‴ 1C–C‴). As retinoid signalling is required for transcription of differentiation genes (including that of *Pax6*) we analysed chromatin organisation around loci in the *Raldh2−/−* mutant embryo, which is unable to synthesize RA in the elongating embryonic body axis [30]. In retinoid deficient embryos caudal FGF signalling expands rostrally from the stem zone [3], [6] and by blocking FGFR signalling in *Raldh2* mutants we dissected the consequences of FGF loss in a context in which many differentiation genes fail to be transcribed. Our data demonstrate, for the first time, that FGF signalling acts upstream of mechanisms that direct higher-order chromatin organisation around differentiation gene loci and further reveal that such mechanisms can be uncoupled from the machinery that mediates transcription of such genes.

## Results

### Chromatin decompacts around the *Pax6* locus coincident with its transcription

**Figure 2:**
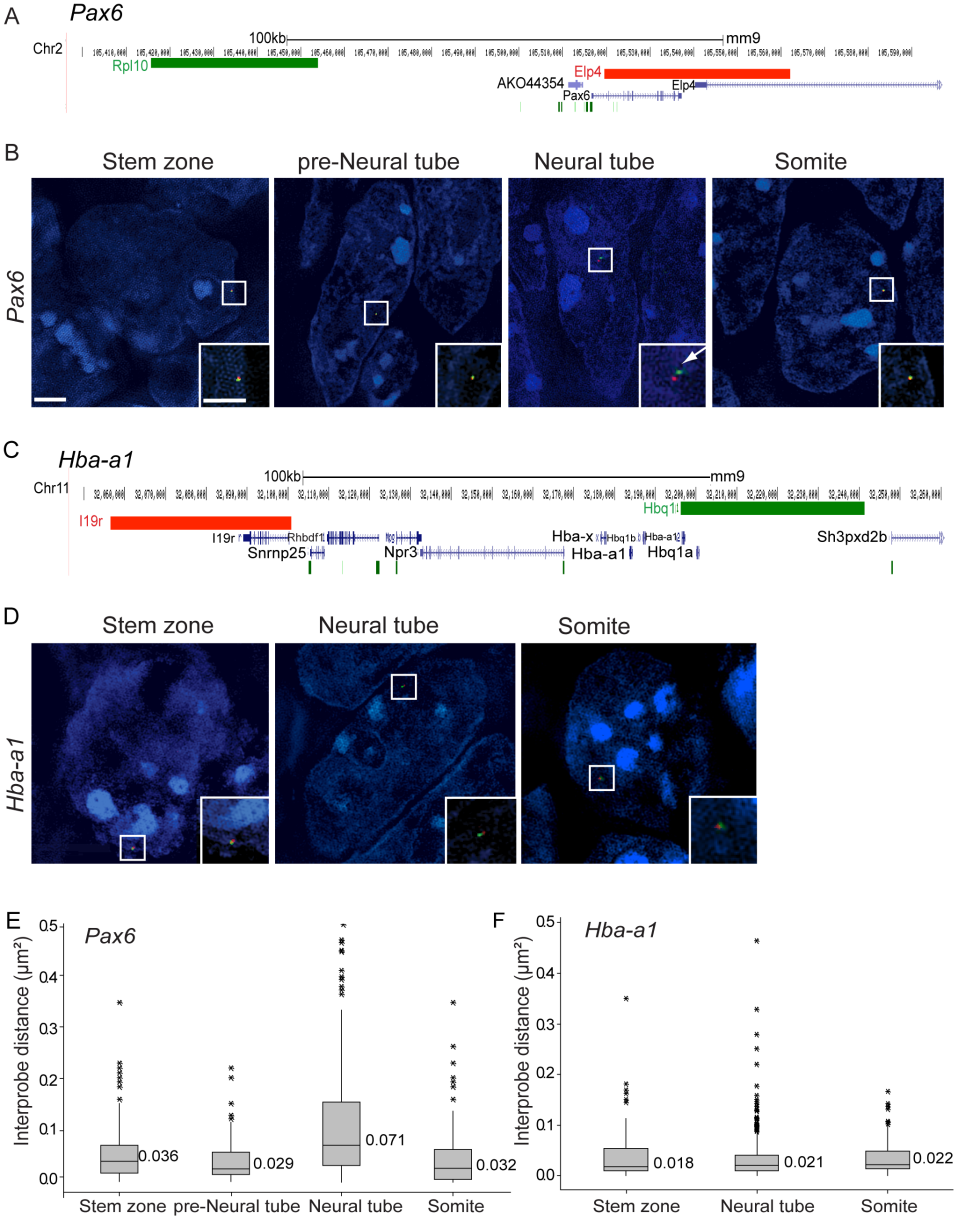
*Pax6* locus decompaction coincides with *Pax6* transcription. (A) Fosmids (green and red bars) used to analyse chromatin around the *Pax6* locus mapped to the mm9 assembly of the mouse genome; (B) Examples of FISH images in DAPI-stained nuclei for the *Pax6*-flanking probe pairs in stem zone, preneural tube, neural tube, and somite. Insets are enlargements of white boxed areas in each image; (C) Fosmids used to analyse chromatin around the control Hba-a1 locus mapped to mm9; (D) Examples of FISH images in DAPI-stained nuclei for the *Hba-a1*-flanking probe pairs in stem zone, neural tube, and somite; (E and F) Box-plots of inter-probe distances (µm^2^) for *Pax6* (E) and control *Hba-a1* (F) flanking probes in each tissue assessed. Scale bar = 2 microns in examples of FISH images and 1 micron in insets, here and in all subsequent figures.

To determine if the onset of *Pax6* transcription in the E8.5 mouse embryo involves a change in chromatin compaction, fosmid probes separated by 65 kb and specific for sequences flanking *Pax6* (Table S1) were used for 3D FISH on wildtype CD1 mouse embryos (Figure 2A). Images were captured using SIM and inter-probe distances, (d) in µm, were measured in transverse sections of the stem zone and preneural tube (which lack *Pax6* transcription) and in the neural tube, where *Pax6* is now transcribed (excluding *Pax6* negative cells in dorsal and ventral most positions) (Figures 1B–B′, 2A, B). Fosmids were also used to measure changes around a control locus, alpha-globin (*Hba-a1*), which is not transcribed in the embryo at this stage [31] (Figures 2C,D). Chromatin compaction was assessed by a comparison of *d^2^* values for each data set, as this is the value that scales linearly with genomic separation [32] and that has been used previously to identify differences in chromatin compaction between cells at different stages of differentiation [24] and between wildtype and mutant cells [13] (Figure 2E) (see Methods for data set collection). There was no statistical difference between *d^2^* values for stem zone and preneural tube (p>0.05), but a clear increase in inter-probe distances across *Pax6* in neural tube in comparison with measurements in either stem zone or preneural tube nuclei (p<0.05, Figures 2B,E, Tables S2, S3). Additionally, in recently formed somites, which lie adjacent to the neural tube and which do not and will not express *Pax6*, chromatin across the *Pax6* locus is as compact as it is in the stem zone and preneural tube, and significantly more compact than in neural tube (Figures 2B, E). In contrast, inter-probe distances around a control gene locus (alpha-globin, *Hba-a1*) were not significantly different between stem zone, neural tube and somite data sets (p>0.05, Figures 2D, F). This controls for any overall change in chromatin condensation at the onset of differentiation. These data therefore indicate that chromatin is compact around the *Pax6* locus in cells that do not and will not express this gene (somites) and those that will later come to express it (stem zone and preneural tube), and that it specifically decompacts coincident with onset of *Pax6* transcription in the neural tube. Analysis of genome-wide histone modification data sets in mouse ES cells and derived neural progenitors [11] further reveals that the *Pax6* locus is subject to H3K27me3 enrichment in ES cells and is relieved of this mark upon neural differentiation, suggesting that this locus is a target of polycomb mediated repression (Figure S1).

We further assessed chromatin organisation across the locus of the gene *Fgf8*, which is expressed in the stem zone and downregulated as neural differentiation commences. *Fgf8* is marked by H3K27me3 and H3K4me3 in mES cells and upon neural differentiation H3K4me3 is lost and H3K27me3 retained [11] (Figure S1). *Fgf8* is a smaller gene than *Pax6* and FISH signals from fosmids flanking this locus (100 kb separation) were barely resolved in any tissue assessed (Figure S2). These findings suggest that polycomb group proteins regulate *Fgf8* expression, but in a manner that does not involve visibly detectable chromatin compaction. Neighbouring genes *Npm3* and *Mgea5* show similar patterns of gene expression as *Fgf8* (Figure S3), but not of histone modifications (Figure S1). This suggests that PRC-mediated chromatin compaction around the *Fgf8* locus may be too subtle, or masked by the chromatin environments of neighbouring genes, to be detected by FISH.

### Nuclear position correlates with transcription of *Pax6* and *Fgf8*

**Figure 3:**
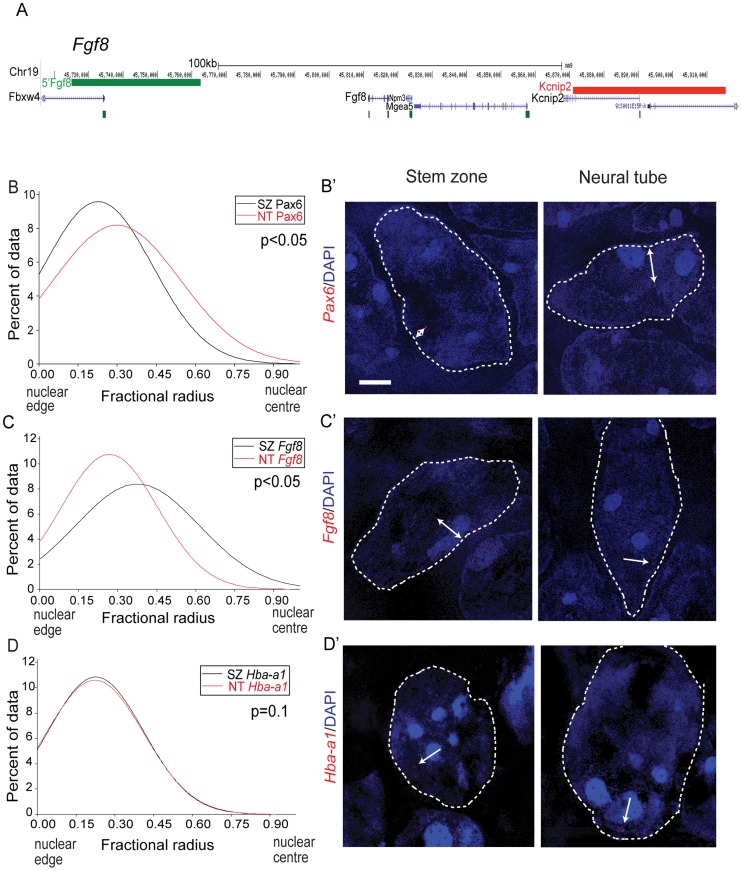
*Pax6* and *Fgf8* loci exhibit altered nuclear radial position coincident with transcriptional status. (A) Fosmids flanking the *Fgf8* locus mapped to the mm9 assembly of the mouse genome; (B) Distribution of fractional radius measurements of the *Pax6* locus (*Elp4* fosmid probe) with respect to the nuclear edge in stem zone (SZ) (black solid line) and neural tube (NT) (red solid line) nuclei. Data are from >50 nuclei per region in each of 3 different embryos; (B′) examples of hybridised nuclei in stem zone and neural tube; (C) Distribution of fractional radius measurements from the *Fgf8* locus (*Kcnip2* fosmid probe) with respect to the nuclear edge in stem zone and neural tube, showing shift towards nuclear periphery in NT; (C′) examples of hybridised nuclei in stem zone and neural tube; (D) Distribution for fractional radius measurements of control locus *Hba-a1* with respect to the nuclear edge in stem zone and neural tube, showing no significant change; (D′) examples of hybridised nuclei in stem zone and neural tube.

To investigate the potential relationship between the position of a gene within the nucleus and its transcriptional activity along the embryonic body axis, we analysed the proximity of FISH signals for *Pax6* and or *Fgf8* (Figure 3A) to the nuclear periphery as defined by DAPI staining. The *Pax6* locus is closer to the nuclear periphery in the stem zone than in the neural tube (p<0.05; Figures 3B,B′), whereas the converse is the case for *Fgf8* (Figures 3C,C′). The relative nuclear position of the control *Hba-a1* locus was the same in the stem zone and neural tube (Figures 3D, D′). These data show that nuclear position correlates well with transcription of *Pax6* and *Fgf8* in the normal embryo.

### Retinoid signalling controls *Pax6* chromatin compaction and nuclear localisation

**Figure 4:**
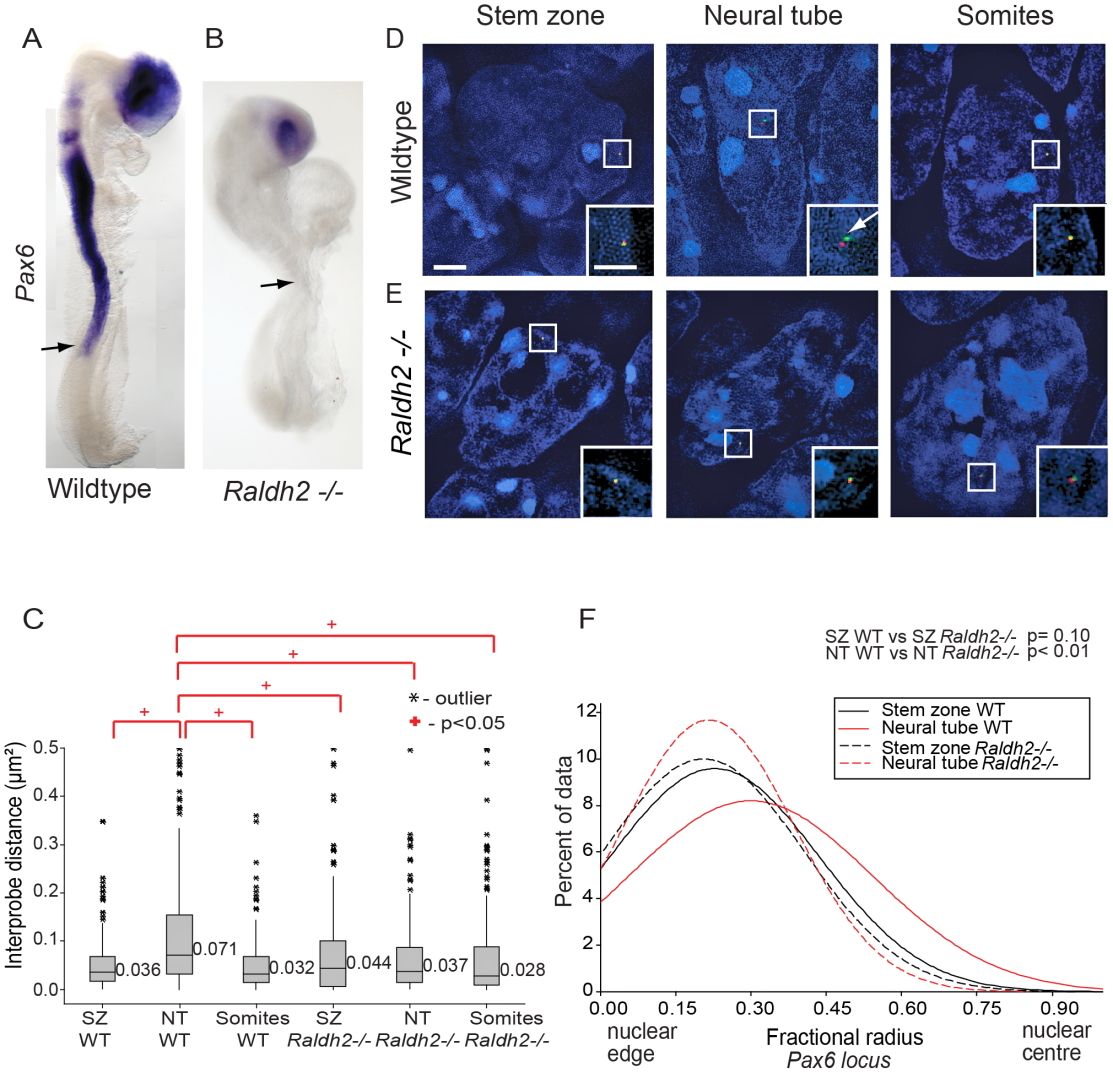
Retinoid signalling is required for decompaction around *Pax6*. (A) *Pax6* is transcribed in wildtype (WT) (B), but not in *Raldh2* mutant neural tube (arrows indicate the last formed somite); (C) Box-plots of inter-probe FISH probe distances, in WT and *Raldh2^−/−^ embryos* showing that in contrast to WT, the *Pax6* locus does not decompact in *Raldh2* mutant neural tube (NT) and distances remain similar to WT stem zone (SZ) and to somites (S); examples of hybridised nuclei in WT (D) stem zone, neural tube and somites, and in *Raldh2* mutant tissues (E) stem zone, neural tube and somites. (F) Graph of data distribution for fractional radius measurements in WT and *Raldh2* mutant tissues, showing that the *Pax6* locus fails to shift towards the nuclear centre in the neural tube in retinoid deficient conditions.

**Figure 5:**
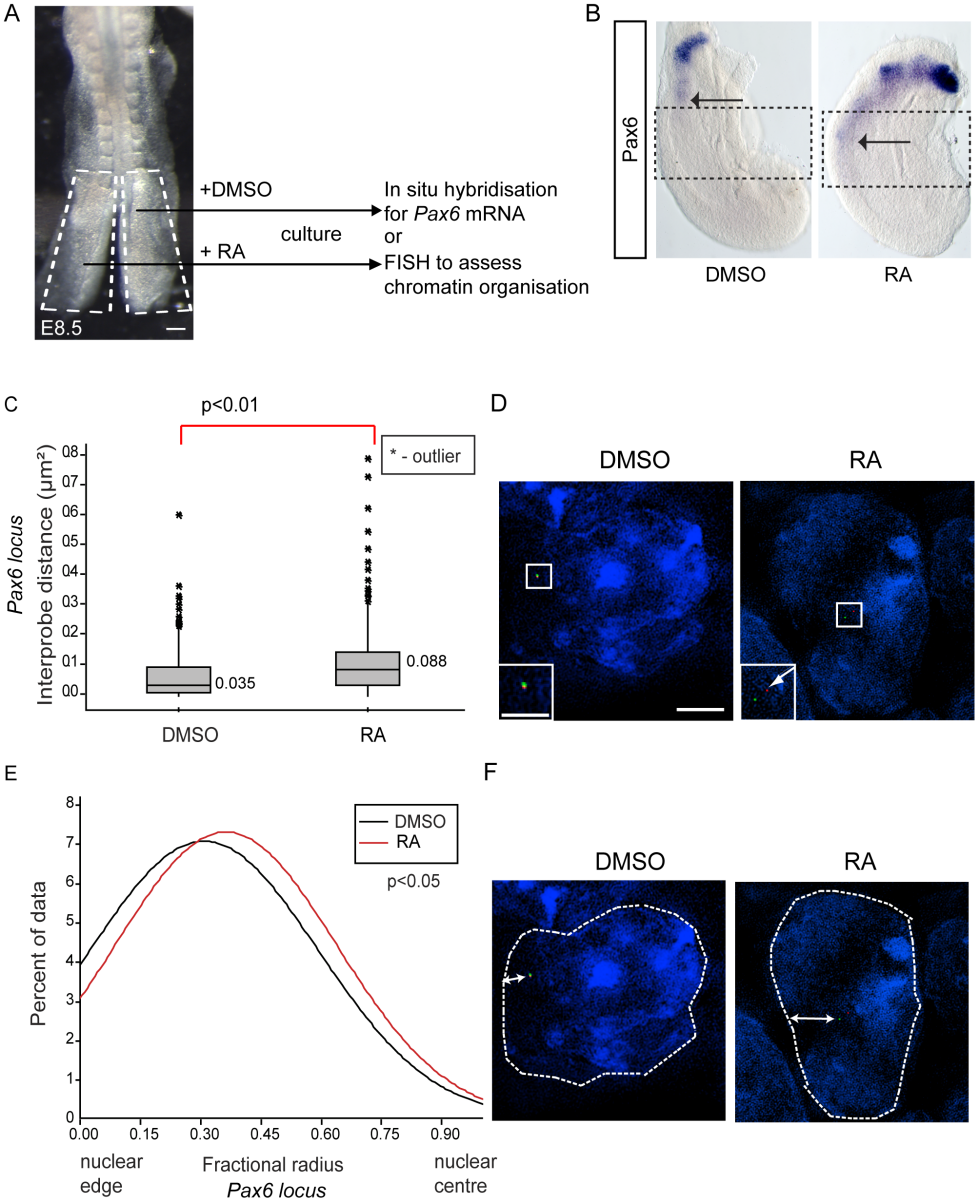
Exogenous retinoic acid induces *Pax6* expression, decompaction and centralised location of *Pax6* locus in stem zone explants. (A) Experimental design, E8.5 embryo bisected along the caudal midline to give an explant pair (white dashed outline), one explant exposed to retinoic acid (RA), the other only to vehicle control (DMSO), followed by analysis for mRNA or chromatin organisation; scale bar = 100 microns (B) Explant pair treated with DMSO or RA analysed for *Pax6* expression (caudal limit of expression indicated by black arrows), dotted lines indicate regions examined by FISH for *Pax6* inter-probe distance; (C) Inter-probe distances across the *Pax6* locus in nuclei taken from sections in the middle third of explants (see Materials and Methods), increased significantly on RA treatment; (D) DAPI stained nuclei and fosmids across the *Pax6* locus from explants following exposure to DMSO or RA; (E) Fractional radius measurements (see Materials and Methods) show shift towards nuclear centre following exposure to RA; (F) DAPI stained nuclei and fosmids across the *Pax6* locus from explants following exposure to DMSO or RA, showing distance from nuclear edge.

*Pax6* is not expressed in the neural tube of mouse embryos lacking the RA synthesising enzyme retinaldehyde dehydrogenase 2 (*Raldh2−/−*) [9], [30] (Figures 4A, B). To determine whether this is also accompanied by failure to undergo changes in higher order chromatin organisation, FISH with probe pairs across the *Pax6* locus was carried out on E8.5 *Raldh2−/−* embryos. There was no difference in chromatin compaction (d^2^) between stem zone and neural tube (p>0.05; Figure 5C) indicating that chromatin decompaction, normally observed across the *Pax6* locus in the wildtype neural tube (Figures 2A, B), does not take place in this retinoid deficient condition. Indeed, the distribution of inter-probe distances in *Raldh2−/−* neural tube nuclei was similar to that found in stem zone of wildtype mice (p>0.05; Figure 4C). The *Pax6* locus also remained compact in somites of wildtype and mutant animals (Figures 4 C, D, E).

The absence of retinoid signalling also resulted in a failure of *Pax6* to reposition away from the nuclear periphery in the neural tube compared to stem zone (Figure 4F). Moreover, *Pax6* is more peripherally located in the *Raldh2*−/− neural tube than in the wildtype neural tube (p<0.05; Figures 4D, E and F). These data show that, for the *Pax6* locus, neither chromatin decompaction nor a shift away from the nuclear periphery take place in the retinoid deficient neural tube in which *Pax6* is not transcribed.

To determine whether exposure to retinoic acid leads to decompaction and a more central nuclear position of the *Pax6* locus we treated explanted caudal regions with retinoic acid or vehicle DMSO control for 10 h (Figure 5A). Explants were then processed either for in situ hybridisation to monitor *Pax6* transcription or for FISH to assess local chromatin organisation (Figure 5B). This confirmed that retinoic acid induces *Pax6* expression (Figures 5B, B′) and demonstrated that this correlates with the decompaction and more central nuclear location of this locus (Figures 5C, D; p<0.05 and p<0.05, respectively).

### FGF signalling regulates chromatin compaction and nuclear position of the *Pax6* locus

FGF signalling ectopically persists in the preneural tube of retinoid deficient quail embryos [3] and in the neural tube of *Raldh2−/−* mouse embryos [5], [6]. As FGF signalling represses onset of expression of neural differentiation genes, including *Pax6*, in the elongating body axis [3], [33], it is possible that failure to express *Pax6* in the *Raldh2* mutant is due to an excess of FGF signalling.

**Figure 6:**
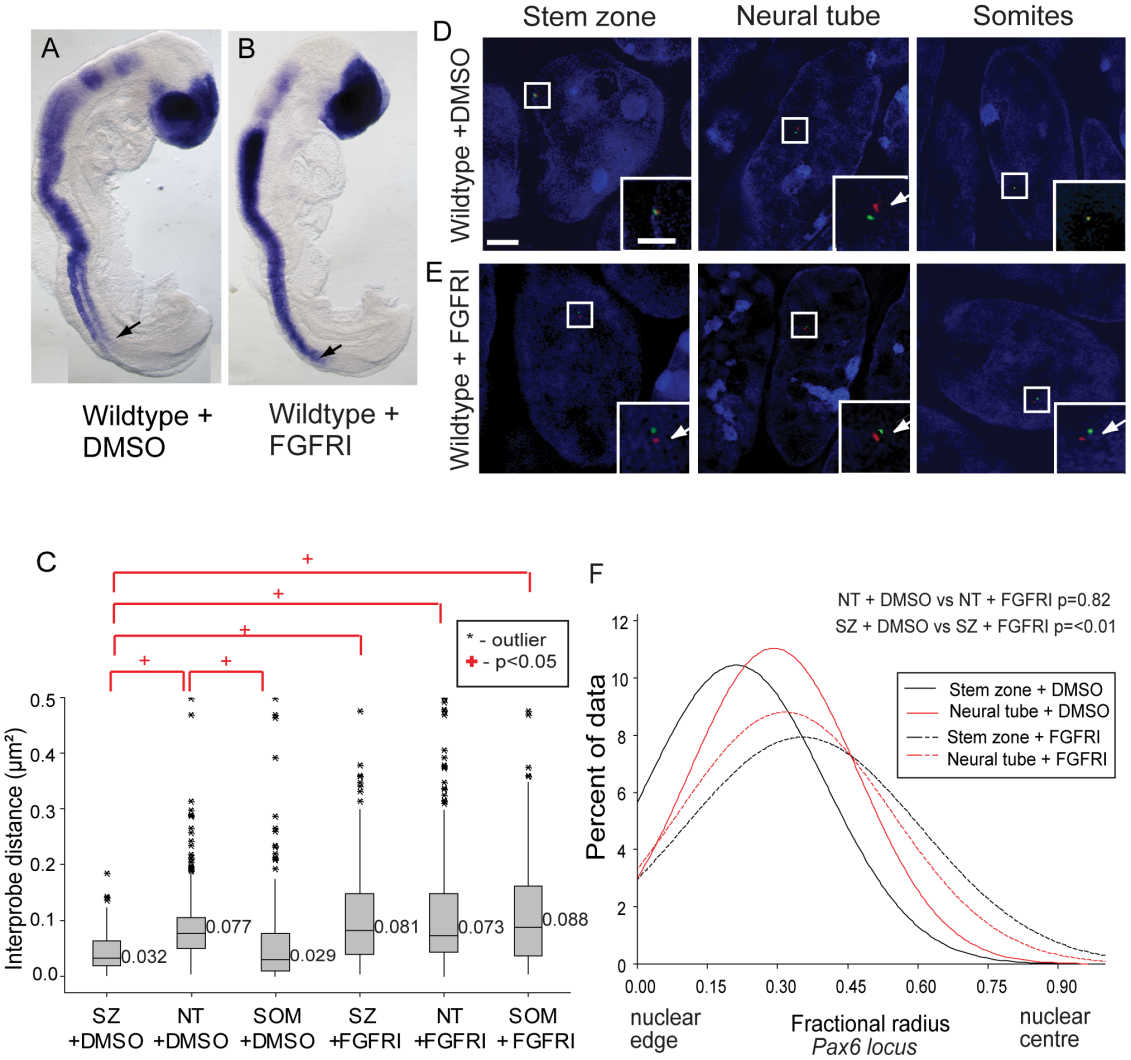
FGF signalling regulates chromatin compaction around *Pax6*. (A) Wildtype (WT) embryos exposed to DMSO or (B) FGFR inhibitor (FGFRI) PD173074, showing that blocking FGF elicits *Pax6* expression in the preneural tube (arrow = last formed somite); (C) Box-plot of inter-probe distances (µm^2^) for *Pax6* flanking probes in each tissue assessed in DMSO and PD173074 treated embryos, showing that FGFR signalling is required to maintain chromatin compaction around the *Pax6* locus in the SZ; examples of hybridised nuclei in DMSO (D) stem zone, neural tube and somites, and PD173074 treated tissues (E) stem zone, neural tube and somites; (F) Graph of data distribution for fractional radius measurements in DMSO and PD173074 treated tissues, showing that the *Pax6* locus now shifts towards the nuclear centre in stem zone as well as in the nucleus of neural tube cells after FGFRI treatment.

To determine whether FGF signalling represses differentiation onset via a mechanism that involves regulation of higher order chromatin organisation, FGF signalling was blocked with the FGFR inhibitor PD173074 [34]. Explanted whole E8 wildtype embryos were cultured *in vitro* exposed to either DMSO vehicle control or PD173074 for 7 h and then processed for FISH, or analysed for expression of the FGFR pathway target *Sprouty2* (*Spry2*) [35] and for *Pax6*. The repression of *Spry2* (DMSO n = 0/5; PD173074 n = 5/5, Figure S4A) confirmed the effective blocking of FGFR signalling (and see [36]). Inhibition of FGFR signalling in the elongating neural axis also leads to precocious onset of *Pax6* expression, which is then detected more caudally in the preneural tube in the chick embryo [33]. Consistent with this, *Pax6* transcripts were detected in the preneural tube of PD173074 treated mouse embryos (DMSO n = 0/4; PD173074 n = 3/4, Figures 6A, B).

Analysis of chromatin compaction across the *Pax6* locus by FISH (Figure 6C) revealed that, unlike the situation in untreated (Figure 2) and control (DMSO treated) embryos where *Pax6* chromatin was more compact (smaller inter-probe distances) in stem zone and somites than in neural tube, this difference was abolished in PD173074-treated embryos. In these conditions the chromatin across *Pax6* appears to decompact in stem zone and the somites to the level normally seen in the neural tube (p<0.05; Figures 6C, D, E). This indicates that FGFR signalling normally promotes chromatin compaction around *Pax6* in caudal regions and somites (see Discussion). Blocking FGFR signalling also promoted a shift in *Pax6* localisation towards the nuclear centre in stem zone and somitic nuclei in comparison with DMSO control (p<0.05 and p<0.05 respectively; Figures 5D, E, and F and S7A). No significant change in compaction or nuclear position in control and PD173074 treated embryos was seen at the control *Hba-a1* locus, indicating that changes in chromatin organisation around the *Pax6* locus do not reflect a general consequence of FGFR inhibition (Figures S4B, C). Together these data indicate that FGF signalling acts upstream of mechanisms that regulate chromatin compaction and nuclear position at *Pax6*.

### FGF signalling regulates chromatin compaction and nuclear position at the locus of a further neural progenitor gene, *Irx3*

**Figure 7:**
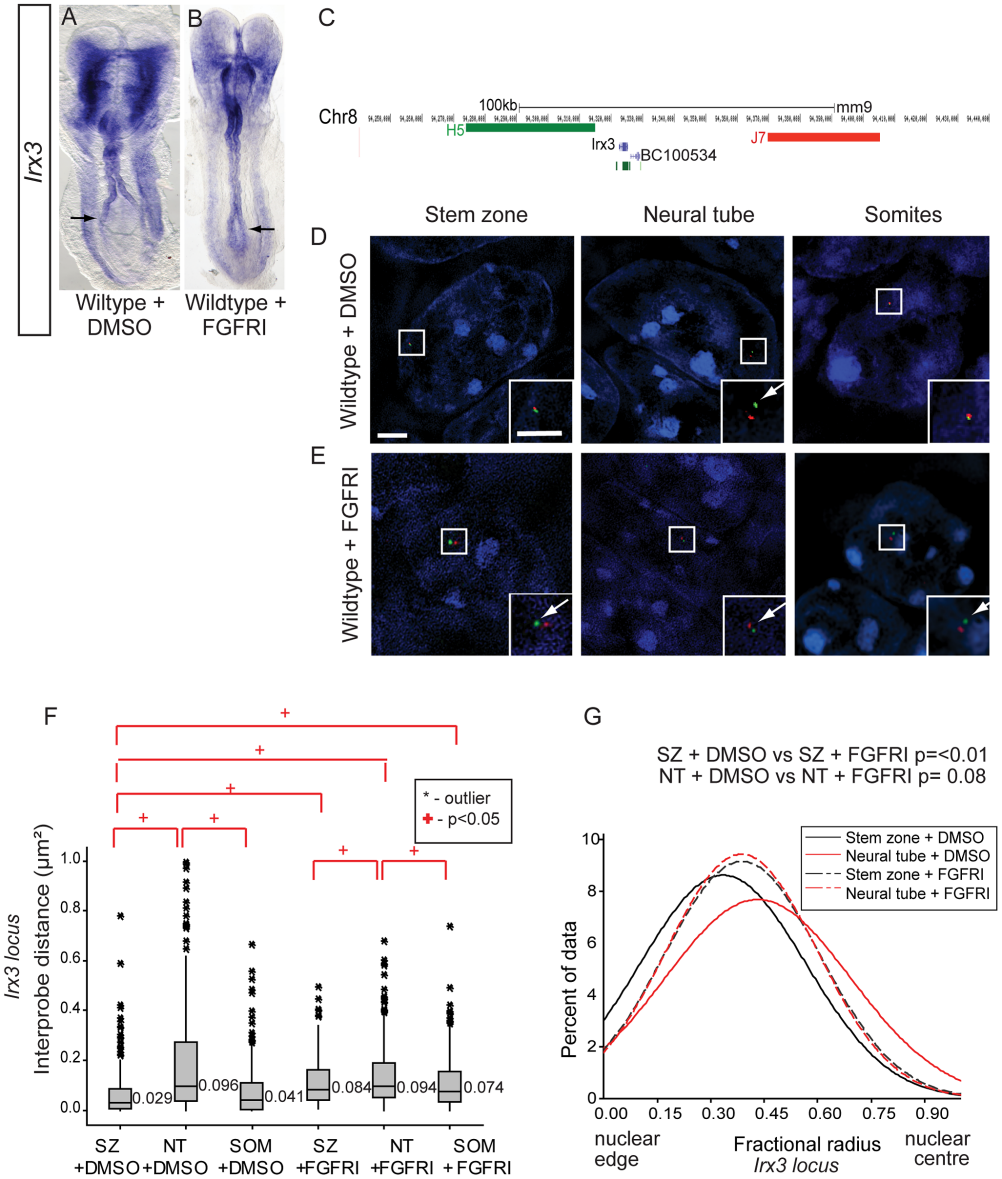
FGF signalling regulates chromatin compaction and nuclear position at the locus of a further neural progenitor gene, *Irx3*. (A) *Irx3* is transcribed in the neural tube of wildtype DMSO treated embryos and (B) its expression extends caudally following exposure to PD173074 for 7 h; (C) Fosmids flanking the *Irx3* locus mapped to mm9; Examples of FISH images in DAPI-stained nuclei for the *Irx3* -flanking probe pairs in stem zone, neural tube, and somite following exposure to (D) DMSO or (E) FGFR inhibitor PD173074; (F) Box-plot of inter-probe distances (µm^2^) for *Irx3* flanking probes in each tissue assessed in DMSO and PD173074 treated embryos, showing that FGFR signalling is required to maintain chromatin compaction around the *Irx3* locus in the stem zone; (G) Graph of data distribution for fractional radius measurements in DMSO and PD173074 treated tissues, showing that the *Irx3* locus now shifts towards the nuclear centre in stem zone as well as in the nucleus of neural tube cells after FGFRI treatment.

To extend this analysis we assessed chromatin organisation around the locus of an additional neural progenitor marker gene, *Irx3*. Like *Pax6*, onset of *Irx3* transcription takes place in the neural tube of the elongating body and is initially broadly expressed across the dorso-ventral axis [37]. Blocking FGFR signalling led to a caudal expansion of the *Irx3* expression domain (n = 0/5 DMSO treated and n = 4/6 PD173074 treated embryos) (Figures 7A, B). Also like *Pax6*, the *Irx3* locus is a PRC target in ES cells [11] (Figure S1). Using fosmids flanking *Irx3* (Figure 7C) FISH analysis confirmed that this region of chromatin decompacts and relocates towards the nuclear centre in the neural tube coincident with its transcription (Figures 7D, E). Furthermore, blocking FGFR signalling led to decompaction and a more central nuclear position of the *Irx3* locus in stem zone nuclei and also somites (Figures 7D, E, S7B). These data demonstrate that FGF signalling consistently acts upstream of chromatin re-organisation at differentiation gene loci.

### Inhibition of FGF signalling in *Raldh2* mutants rescues higher order chromatin organisation, but not transcription, at the *Pax6* locus

**Figure 8:**
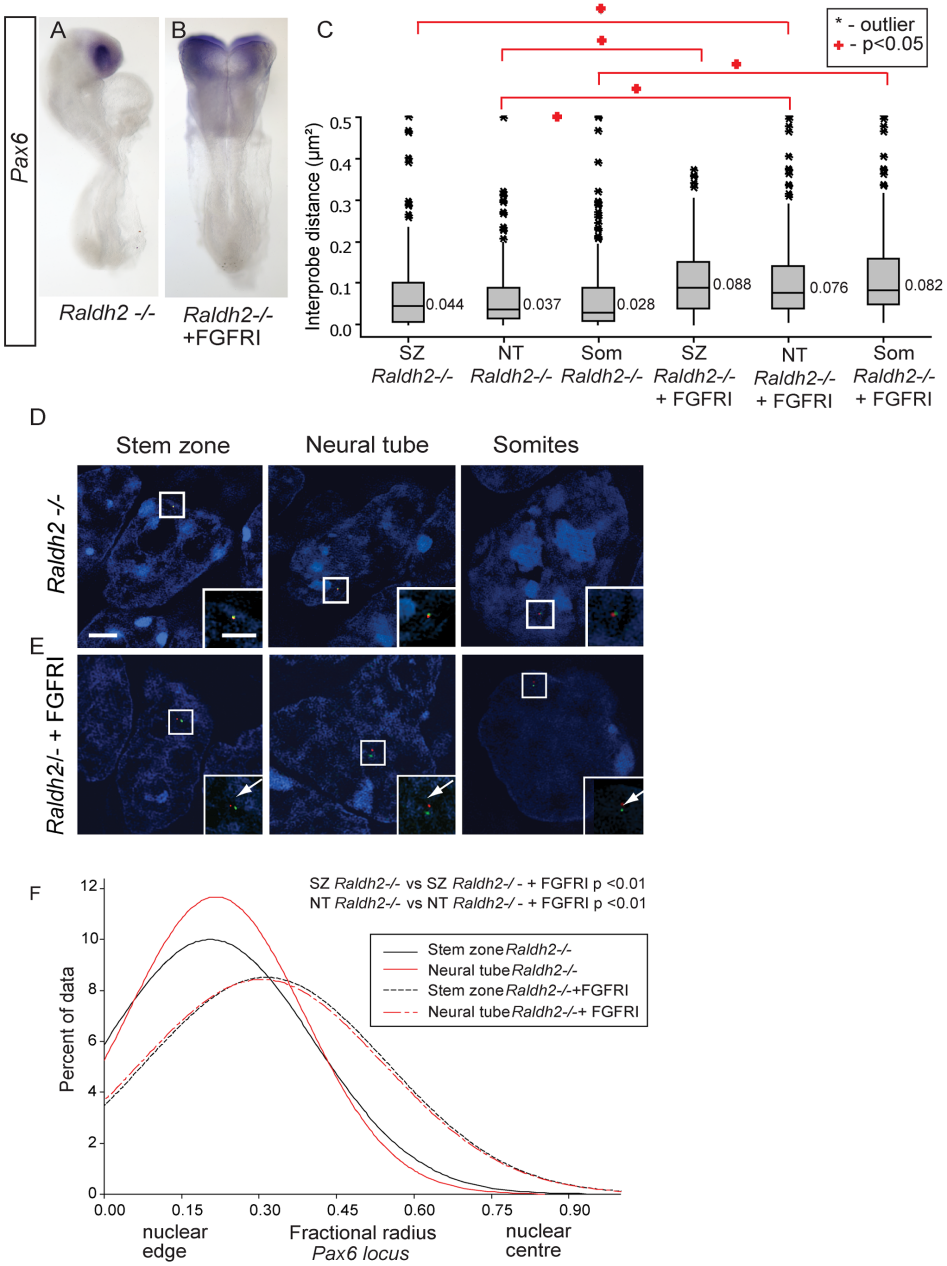
Inhibition of FGFR signalling in *Raldh2* mutants rescues higher order chromatin organisation around the *Pax6* locus, but not *Pax6* transcription. (A) *Pax6* transcripts are lacking in the neural tube of *Raldh2* mutants treated with DMSO or (B) with FGFR inhibitor PD173074; (C) Box-plot of inter-probe distances (µm^2^) for *Pax6* flanking probes in each tissue assessed in *Raldh2* mutant embryos or *Raldh2* mutants treated with PD173074, showing blocking FGFR signalling decompacts the *Pax6* locus in both stem zone, neural tube and somites; and examples of hybridised nuclei in *Raldh2* mutant embryos, D) stem zone, neural tube and somites, or treated with PD173074, (E) stem zone, neural tube and somites. (F) Graph of data distribution for fractional radius measurements in *Raldh2 −/−* and *Raldh2−/−* + PD173074 tissues, showing that the *Pax6* locus now shifts towards the nuclear centre in stem zone as well as in the neural tube when FGFR signalling is blocked.

To determine whether excess of FGF signalling is responsible for the lack of *Pax6* expression in the absence of retinoid signalling, *Raldh2* mutant embryos, and their wild type littermates produced from heterozygous *Raldh2+/−* crosses, were cultured with PD173074 or DMSO. Strikingly, blocking FGFR signalling did not rescue *Pax6* expression in the neural tube of *Raldh2−/−* embryos (*Pax6* mRNA was detected in neural tube of 0/4 mutant embryos and 0/4 PD173074 treated mutant embryos; Figures 8A, B). Attenuation of FGFR signalling in this condition is therefore not sufficient for onset of *Pax6* expression and is consistent with a further requirement for retinoid signalling to promote neural differentiation [3].

This finding does, however, raise the possibility, that blocking FGFR signalling in retinoid deficient conditions still promotes initial steps in the differentiation process upstream of *Pax6* transcription and this perhaps includes chromatin re-organisation. Indeed, FISH revealed that the *Pax6* region decompacts in the stem zone and in the neural tube of PD173074 treated *Raldh2−/−* embryos compared to the control DMSO-treated mutant embryos (p<0.05 for both comparisons; Figures 8C,D, E). Blocking FGFR signalling in wildtype or in *Raldh2* mutant embryos also decompacts chromatin in somites, despite the absence of *Pax6* expression in this tissue (p<0.05 for both comparisons; Figures 8C,D, E) (see below). Similarly, blocking FGFR signalling in this context also induced a shift towards the nuclear centre of the *Pax6* locus in both stem zone and neural tube; and this relative position is similar to that seen in the neural tube of wildtype or DMSO-treated embryos (p>0.05, Figure 8F). In this context, FGFR signalling therefore acts upstream of mechanisms that direct both local chromatin compaction and nuclear position and that can be uncoupled from the activity of retinoid mediated transcription factor complexes that are required to promote expression of neural differentiation genes such as *Pax6*.

### Loss of *Fgf8* peripheral nuclear localisation in retinoid deficient neural tube and its rescue by inhibition of FGFR signalling

**Figure 9:**
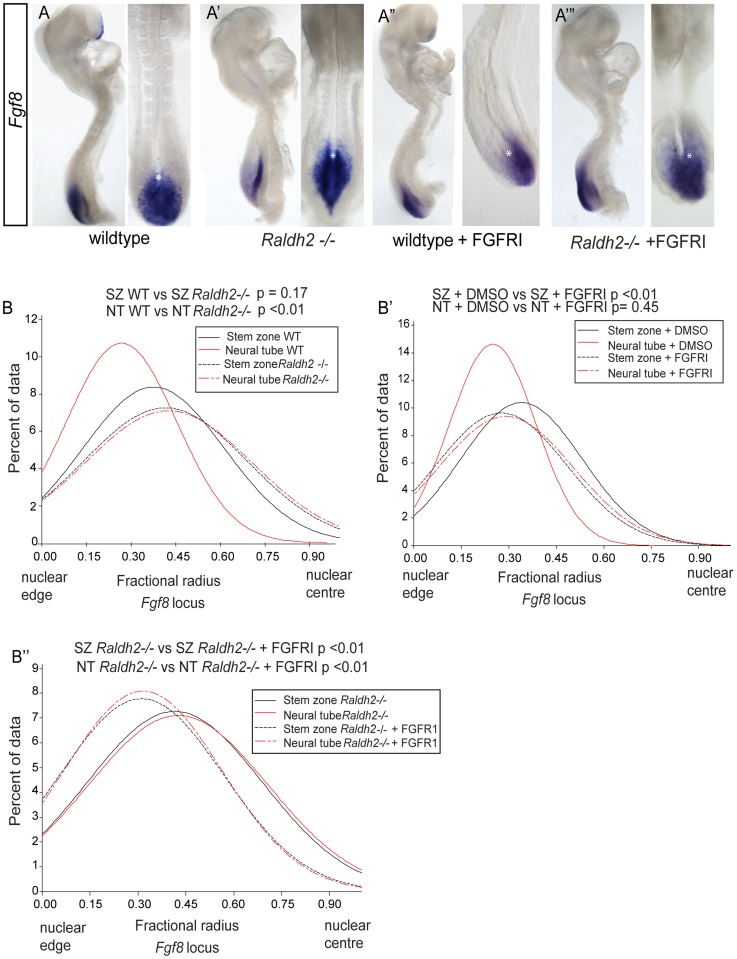
Ectopic *Fgf8* expression in *Raldh2* mutants correlates with a more central nuclear position, which is dependent on FGF signalling. *Fgf8* expression patterns in (A) wildtype, (A′) *Raldh2 −/−*, (A″) wildtype treated with FGFR inhibitor, PD173074, (A‴) *Raldh2−/−* treated with PD173074, asterisk indicates node, note *Fgf8* transcripts extend further rostral in *Raldh2 −/−* embryos; Graphs of data distribution for fractional radius measurements from the *Fgf8* locus in (B) WT vs *Raldh2 −/−* ; (B′) WT DMSO vs FGFRI/PD173074 treated; and (B″) *Raldh2 −/−* vs *Raldh2 −/−* + FGFRI/PD173074 treated embryos (for images for *Fgf8* fosmids in each condition and tissue, see Figure S5).

Although we could not use FISH to measure chromatin compaction at the *Fgf8* locus, this approach can be used to assess nuclear position. Consistent with rostral expansion of *Fgf8* transcription in such mutants [6] (Figures 9A, A″), the *Fgf8* locus fails to locate towards the nuclear periphery in the *Raldh2* −/− neural tube, (p<0.05 in comparison with wildtype, Figures 9B, S5). Here, nuclear position therefore correlates with changing *Fgf8* expression and these findings indicate that retinoid signalling is upstream of mechanism(s) that directs nuclear position of the *Fgf8* locus.

It is further possible that the location of the *Fgf8* locus is influenced by FGF signalling itself, as transcription of *Fgf* genes can be maintained by positive auto-regulatory feedback loops e.g. [38], [39]. To address this possibility we blocked FGFR signalling in wildtype and *Raldh2−/−* mutants (Figures 9A″, A‴, B′, B″). In both conditions this led to a more peripheral localisation of the *Fgf8* locus in the stem zone (where this gene is expressed) in comparison with wildtype and *Raldh2*−/− DMSO controls. Strikingly, in the *Raldh2* −/− mutant neural tube blocking FGFR signalling also rescued the failure to shift to the nuclear periphery observed in untreated mutants (Figures 9B–B″, S5). *Fgf8* transcripts are still detected in PD173074 exposed embryos (Figures 9A″, A‴′) and this may reflect the known stability of *Fgf8* mRNA [40], (although some intronic *Fgf8* transcripts were detected in the stem zone of PD173074 treated embryos by whole mount in situ hybridisation (Figure S6), indicating that not all active *Fgf8* transcription is lost). Overall, these findings demonstrate that FGFR signalling regulates nuclear position of the *Fgf8* locus and that it is responsible for the persistent central location of this gene in the retinoid deficient neural tube.

## Discussion

**Figure 10:**
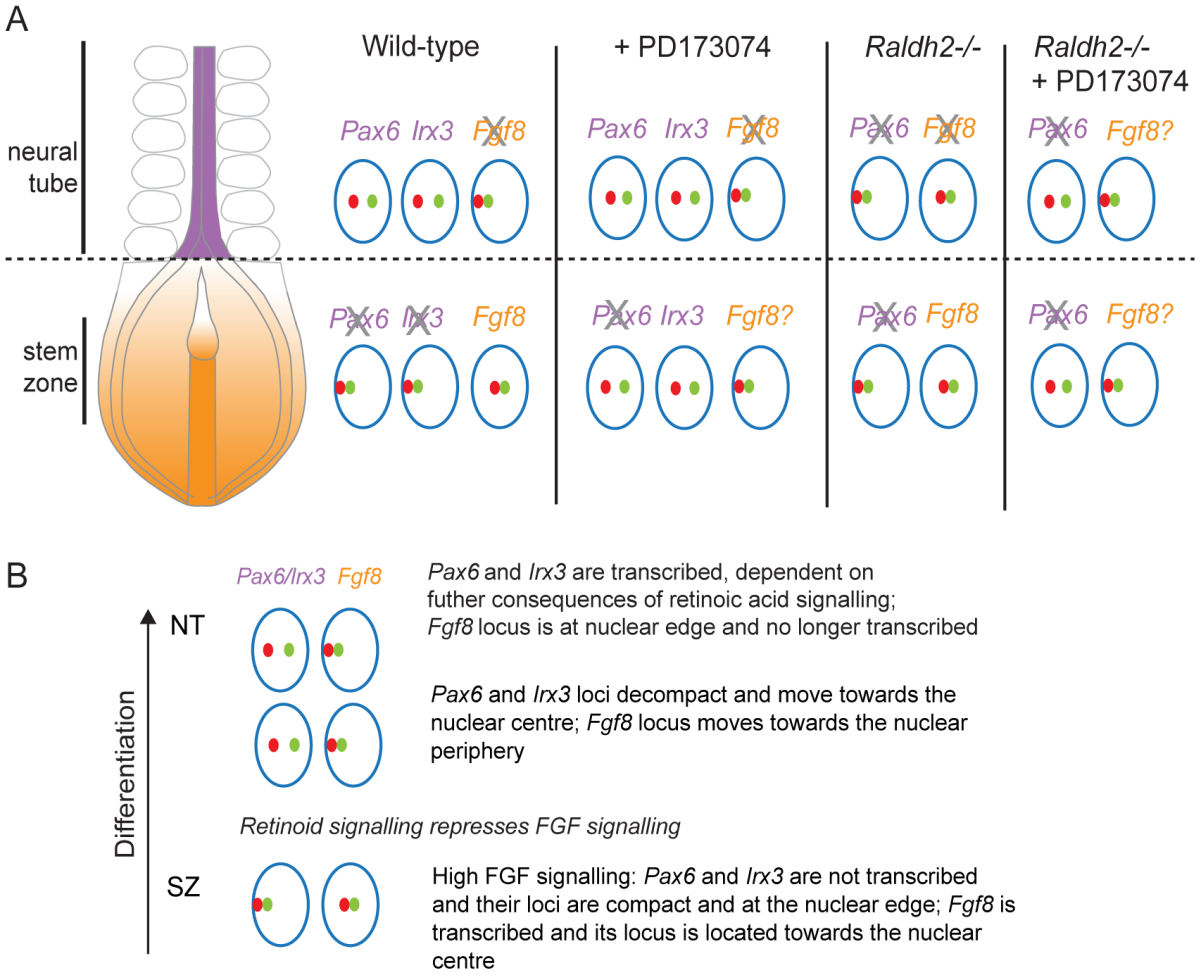
Changes in chromatin compaction and nuclear position of *Pax6* and *Fgf8* loci during neural differentiation and following manipulation of retinoid and/or FGF signalling. (A) Schematic summarising changes in local chromatin organisation of *Pax6, Irx3* and *Fgf8* loci as neural differentiation commences in the elongating body axis of wildtype and FGFR signalling deficient (+PD173074) and for *Pax6* and *Fgf8* in retinoid deficient (*Raldh2−/−*) mouse embryos and when both these signalling pathways are attenuated. These data indicate that FGF signalling promotes chromatin compaction around *Pax6* and *Irx3* loci and regulates nuclear position of *Pax6, Irx3* and *Fgf8* gene loci during neural differentiation. Green and red dots represent flanking fosmid pairs and blue circle the nuclear edge, grey cross indicates likely loss of active *Fgf8*. (B) Summary of chronological steps towards neural differentiation deduced in this study, from the high FGF signalling context in the stem zone to the onset of neural gene expression in the high retinoid signalling environment of the neural tube.

This study reveals changes in higher-order chromatin organisation during neural differentiation in the mouse embryo and demonstrates, for the first time, that this level of organisation is regulated by key signalling pathways that direct differentiation (summarised in Figures 10A, B). We identify FGF signalling in the caudal region of the embryo as a factor acting upstream of mechanisms promoting chromatin compaction and peripheral nuclear position at neural differentiation gene loci, and further demonstrate that these large-scale changes can be uncoupled from transcription. We additionally show that FGF signalling promotes a central nuclear position for the *Fgf8* locus. These data demonstrate that FGF can constrain differentiation via multiple mechanisms that control higher-order chromatin organisation.

### Chromatin compaction changes during neural differentiation

We found that chromatin decompaction around *Pax6 and Irx3* correlate with their transcriptional activation in the newly generated neural axis. The decompaction observed at these loci is reminiscent of that seen upon activation of Hox loci during embryonic development [23], [24], where chromatin compaction of the silent loci has been shown to be mediated by the PRC1 polycomb complex [13]. Decompaction around *Pax6* and *Irx3* in the neural tube nuclei corresponds to the first transcription of these genes during development and is consistent with polycomb regulation as indicated by the presence of H3K27me3 [11] and also the PRC1 protein Ring1b at the *Pax6* locus in ES cells [41]. H3K27me3 is also associated with the *Fgf8* locus in ES cells [11]. As we detect no increase in chromatin compaction when *Fgf8* is transcriptionally downregulated, it is possible that in the embryo this does not involve polycomb mediated repression. However, *Fgf8*, but not neighbouring genes (*NPM3* and *Mgea5*), has associated H3K27me3 in ES cells (Figure S2) and we cannot exclude that compaction local only to *Fgf8* is not detected by our FISH assay.

We found that retinoid signalling is sufficient and necessary for chromatin decompaction around the *Pax6* locus. *Pax6* is not a direct target of the RAR/RXR transcriptional complex, but has an important cross-regulatory relationship with the proneural gene *Ngn2* which is a direct target [8], [42]–[44]. However, RA signalling has been linked directly to the loss of PRC2 binding and H3K27me3 due to its promotion of MSK1/2 mediated phosphorylation of H3S28 in an embryonic carcinoma cell assay [45]. This modification is adjacent to H3K27 and correlates with the loss of PcG binding and loss of repression at a subset of PRC target genes. Nevertheless, RA is just one of several extrinsic signals that can promote MSK1/2 activity *in vitro* and mice null for both MSK1 and 2 are viable and fertile [46], suggesting that regulation of MSK1/2 is not a key endogenous mechanism for removal of polycomb-mediated repression in development. Instead, our data indicate that the requirement for retinoid signalling in the embryo is for removal of FGF signalling, which is in turn responsible for compaction around *Pax6* and *Irx3*.

### Altered nuclear positioning during neural differentiation

As well as undergoing chromatin decompaction, *Pax6* and *Irx3* also relocates to a more central position in the nucleus in the neural tube. For *Pax6*, this does not occur in the absence of retinoid signalling. *Fgf8* shows the converse pattern of nuclear movements, but in the apparent absence of chromatin compaction changes, suggesting that nuclear position and local chromatin organisation are regulated by distinct molecular mechanisms. The relocation of *Fgf8* toward the edge of the nucleus in the neural tube is blocked in *Raldh2* mutants which correlates with its ectopic transcription [6]. *Fgf8* has upstream RAREs indicating that it may be directly repressed by RA signalling [47], [48] and our finding here suggests that this may be linked to nuclear positioning mechanisms.

Importantly, although nuclear position of the *Fgf8* locus correlated well with transcription of this gene, we found no cellular context (including wildtype, FGFR or retinoid signalling deficient conditions) in which chromatin decompaction (observed around *Pax6* and *Irx3*) took place without a concomitant shift towards the nuclear centre. This suggests that de-compaction may be contingent upon a more central nuclear position.

Association with nuclear lamins correlates well with location of genomic regions to the nuclear periphery and so-called Lamin associated domains (LADs) generally have low levels of transcription. Genome-wide maps of Lamin B1 association in mouse ES cells show that many neural differentiation genes are located in LADs [29]. However, neither the *Pax6* nor *Irx3* loci, nor the whole *Fgf8* region is a LAD in either ES cells or ES-derived neural progenitors (Figure S8) suggesting that at least in this *in vitro* context nuclear re-positioning of these loci is unlikely to be mediated by altered Lamin B1 association. However, while Lamin B genes appear not to be required for differentiation in ES cells, Lamin B null mice do exhibit profound neural defects [49], [50]. These include both neural progenitor proliferation and nuclear lamina integrity, and so a role for LAD mediated regulation of nuclear positioning of neural differentiation genes in the embryo cannot be ruled out [49], [50].

### FGF signalling is upstream of higher-order chromatin organisation

Our discovery that blocking FGFR signalling in the *Raldh2* mutant, where many differentiation genes fail to be transcribed, restores chromatin decompaction and a more central nuclear position of the *Pax6* locus suggests that RA acts first to inhibit FGFR signalling and that FGF is upstream of molecular mechanism(s) that direct higher order chromatin organisation at differentiation gene loci. This result is further supported in wildtype embryos in which FGFR signalling is blocked, as here *Pax6* transcription extends caudally, being precociously expressed in the preneural tube, but we detect *Pax6* decompaction and its more central nuclear position in stem zone cells, which have yet to express *Pax6*. Importantly, these experiments uncouple regulation of higher order chromatin organisation from gene expression itself and indicate that these large-scale chromatin changes take place as an initial step in the differentiation process. Although we assess these changes by detailed investigation around two exemplar neural differentiation genes, FGF signalling in this context represses expression of many such genes, including *Sox1* and *Sox3* (further regulators of the neural progenitor cell state) and prevents the onset of ventral patterning and neuron production in the newly generated spinal cord [3], [7]. It is therefore likely that FGF signalling (directly or indirectly) regulates a general mechanism(s) that determines chromatin organisation at such differentiation genes, many of which are known PRC2 targets in ES cells.

Intriguingly, blocking FGFR signalling also led to decompaction and a more central nuclear position of *Pax6* and *Irx3* loci in somites, where this gene is never expressed. These somites will have formed (1 somite every 2 hours) during the 7 h period of exposure to FGFR inhibitor, at the start of which these cells would have been experiencing FGF signalling in the presomitic mesoderm. The reorganisation of *Pax6* and *Irx3* loci in this context may thus reflect the finding that high level FGF signalling is required for mesoderm induction, while reduction elicits neural differentiation, as observed in *Fgfr1* mutant mice, reviewed in [1]. Sudden loss of FGFR signalling in the early presomitic mesoderm might therefore elicit initial steps in neural differentiation.

Importantly, we show that blocking FGFR signalling does not lead to global chromatin reorganisation, as inter-probe distances and the fractional radius for control *Hba-a1* locus and inter-probe distance for the region of the *Fgf8* locus remain unchanged in all tissues examined. The *Fgf8* locus does, however, alter its nuclear position in response to changes in FGFR signalling. When FGFR signalling is blocked in either wildtype or *Raldh2* mutant embryos the *Fgf8* locus remains close to the nuclear periphery in all tissues examined, including the stem zone where this gene is normally expressed and in the *Raldh2 −/−* mutant, where this inhibition of FGF signalling rescues the ectopic centralised location of *Fgf8* locus. Although location at the nuclear periphery generally correlates with gene repression, we do detect some intronic *Fgf8* transcripts in PD173074 treated embryos, indicating that in the timeframe of this experiment the peripheralisation of the *Fgf8* locus does not simply correlate with loss of transcription. This may reflect an initial heterogeneous response to the loss of FGF signalling across the stem zone cell population, however, active transcription and peripheral locus position it is not incompatible with transcription [27], [28]. Overall then, FGF is upstream of mechanisms in the stem zone that lead to *Pax6* and *Irx3* compaction and peripheral location, and that promote a central position of *Fgf8* within the nucleus. This shows that in this context FGF signalling influences multiple distinct molecular mechanisms, which regulate chromatin compaction and promote movement towards or away from the nuclear centre in a locus specific manner.

Attenuation of FGF signalling in human embryonic stem (hES) cells and mouse epiblast stem cells leads to loss of self-renewal [51]–[53]. Furthermore, as observed in the elongating embryonic neural axis [33] and in mouse ES cells that have experienced a period of endogenous FGF/Erk [7], inhibition of FGF/Erk signalling in hES cells induces rapid expression of *Pax6* [53]. The attenuation of FGF signalling in stem cells of epiblast origin and in multipotent epiblast cells located in the stem zone/caudal lateral epiblast therefore serves as a common trigger for onset of differentiation and it is likely that conserved molecular mechanisms that include relief from polycomb mediated repression at differentiation genes underlie this initial step. Key future tasks are to determine how FGF signalling regulates local chromatin compaction and orchestrates nuclear positioning to constrain cell differentiation.

## Materials and Methods

### Mouse embryo collection, culture and exposure to small molecules

Wildtype CD1 embryos were collected at E8.5, dissected, fixed and processed for *in situ* hybridization (ISH) or for FISH as described below. Heterozygous *Raldh2* mutant CD1 mice [30] were crossed to generate litters at E8-8.5 containing *Raldh2−/−, Raldh2+/−* and wildtype embryos. These were either dissected, genotyped as described previously [30], fixed and processed for ISH or FISH (see below), or E8 embryos within yolk sacs were collected in warmed (37°C) culture medium (rat serum, tyrode solution; 1∶1) containing control DMSO (0.5 µl/1 ml culture medium) or FGFR inhibitor PD173074 (Calbiochem) at 50 µM. Embryos were then cultured for 7 hours in a water-saturated roller-tube incubator at 37°C in 5% CO_2_, 20% O_2_. These were then dissected, genotyped, fixed and processed for FISH. For treatment with retinoic acid wild type CD1 E8-8.5 embryos were dissected to give explants pairs of the caudal embryo (Figure 5A) with one explant treated with 250 nM RA and the other DMSO vehicle control cultured in collagen as previously described [3] for 10 h. Explants were then fixed in 4% PFA and processed for ISH or FISH. For FISH analysis nuclei in sections taken from the central third of each explant were measured (5 explant pairs, >30 nuclei per explant measured) for inter-probe distance and fractional radius. Initial analyses compared differences between treated and untreated explants taken from the same embryo and these were all significantly different (Table S4). We therefore pooled all treated and all untreated explant data (Figures 5C, E).

### Ethics statement

All procedures using animals were performed in accordance with UK and French legislation and guidance on animal use in bioscience research.

### In situ hybridisation for mRNA

Standard procedures were used to carry out in situ hybridisation in whole embryos to detect mRNAs for *Pax6, Irx3, Fgf8, Spry2, Npm3* and *Mgea5/OGA* (primers used to clone *Irx3*, *Npm3* and *Mgea5/OGA* can be found in Figure S3). A subset of these were embedded and cryo-sectioned to visualise mRNA localisation at a cellular level. Intronic *Fgf8* was detected using a probe for the region between exons 5 and 6 of the mouse *Fgf8* gene (a kind gift from Olivier Pourquie, [40]).

### Fluorescence DNA in situ hybridisation

Mouse embryos stored in 100% MeOH were cleared in xylene, embedded in wax, sectioned at 7 microns and dried down on thin TESPA-coated 50×22 #1.5 coverslips (Scientific Laboratory Supplies Ltd) suitable for OMX microscopy. The protocol for FISH on mouse tissue sections was then adapted from [54]. Coverslips with sections were heated to 65°C (20 min), washed ×4 in xylene (10 min) and re-hydrated through an ethanol series to dH_2_0. Coverslips were then microwaved for 20 min in 0.1 M citrate buffer, pH6.0, cooled in buffer (20 min) washed and stored in dH_2_0 prior to pre-hybridisation steps and denaturation as previously described [54]. Fosmids pairs separated by inter-genomic distance of 60–120 kb were selected from the WIBR-1 Mouse Fosmid Library (Whitehead Institute/MIT Center for Genomic Research) and sequences confirmed by targeted PCR (Table S1, Figure S9). These were then labelled with either digoxigenin-11-dUTP or biotin-16-dUTP by nick transcription. Approximately 150 ng probe along with 15 µg mouse C*ot*1 DNA (Invitrogen) and 5 µg sonicated salmon sperm DNA (sssDNA) were used per coverslip, denatured and hybridised to coverslips [54]. After overnight incubation and washing, digoxigenin labelled probes were detected with anti-dig FITC (1∶20, Roche) and amplified with anti-sheep Alexa Fluor 488 (1∶100, Molecular Probes); biotin labelled probes with biotinylated anti-avidin (1∶100) and Alexa streptavidin 594 (1∶500, Molecular Probes). Nuclei were counterstained with DAPI and coverslips mounted onto slides with 25 µl of Slowfade Gold (Molecular Probes).

### Structured illumination image acquisition and processing

Samples were imaged on a Deltavision 3D OMX Structured Illumination Microscope (Applied Precision) using a protocol adapted after [55]. Regions of interest (ROIs) were identified using a Deltavision microscope, mapped using Softworx (Applied Precision) and acquired with a UPlanSApochromat 100× 1.4 NA oil-immersion objective lens (Olympus) and back-illuminated Cascade II 512×512 EMCCD camera (Photometrics) on the OMX version 2 system (Applied Precision) equipped with 405, 488, and 593 solid-state lasers. Samples were illuminated by a coherent scrambled laser light source that had passed through a diffraction grating to generate the structured illumination. Potential photo-bleaching was minimised by using lowest possible laser power and exposure times (50 and 250 ms). Raw images were processed and reconstructed using the Softworx structured illumination reconstruction tool (Applied Precision) [56]. The 405, 488 and 593 channels were then aligned in *x* and *y*, using predetermined shifts which were measured using a target lens and 100-nm Tetraspeck fluorescent beads (Invitrogen) in the Softworx alignment tool (Applied Precision).

### Image analysis for chromatin compaction and nuclear position

For analysis of chromatin compaction and nuclear position, measurements were made in images of >50 nuclei per region in each of 3 different embryos per condition.

Stem zone was defined as epiblast cells adjacent and just caudal to the node (∼5 sections per embryo), preneural tube as neuroepithelium rostral to the node underlain by notochord and presomitic mesoderm, neural tube as neuroepithelium flanked by 2 or 3 most recently formed somites, and these adjacent somites were also used to represent somitic tissue. As nuclei in tissues are not as spherical as in cultured cells it was not possible to apply standard nuclear segmentation tools to define nuclear position. Instead sections in which a fosmid signal and nuclear edge were in sharp focus were used to measure the shortest distance from the probe centre to the periphery. The broadest distance across the nucleus was also measured as an indication of nuclear diameter and this was halved and data presented as a proportion the nuclear radius (fractional radius). Super resolution images were uploaded into an OMERO server (Open Microscopy Environment) and ROIs containing hybridisation signals for both dig and biotin-labelled probes were identified by manual inspection in OMERO-insight. ROIs typically extended over several z-sections to accommodate the whole volume of the signals. These ROIs were analysed by a custom script developed in MATLAB (Michael Porter, University of Dundee). This script first segments the objects defined by each probe from the background using Otsu thresholding and then calculates the xyz coordinates the centroid in each object. The centroids of these two objects and the distance between them, d (µm), were then output to a spread-sheet. The inter-probe distance was then squared because in interphase nuclei the mean physical distance squared between two points is linearly related to the known genomic distance [32]. Within each nucleus, the line measurement tool was used to determine the distance of the edge of the nucleus from the hybridisation signal of the biotin-labelled probe in sections in which it was in sharp focus and this was then averaged. The radius of the nucleus, also measured with the line measurement tool, was then divided by this distance. This gave the distance of the gene locus from the nuclear periphery as a proportion of nuclear size.

### Statistical analysis

Box plots in figures show distribution of data. Top and bottom whiskers show highest and lowest data points respectively. Top and bottom lines of box represent 3^rd^ and 1^st^ inter-quartiles and the middle line represents the median. Non-parametric Mann-Whitney U test was used for analyses as data were not normally distributed. For comparison between explant pairs, a paired-sample Wilcoxon signed-rank test was used (Table S4).

## Supporting Information

Figure S1 Histone modifications around key loci in ES cells and derived neural progenitors. Genomic co-ordinates are from the mm8 assembly of the mouse genome and histone modifications indicative of active H3K4me3 and silenced H3K27me3 regions around *Pax6* (A), *Irx3* (B), *Fgf8* (C) and *Hba-a1* (D) in mouse embryonic stem (ES) cells and ES cell derived neural progenitors (NP) (from dataset of [11]).
(TIF)

Figure S2 Assessment of chromatin compaction at the *Fgf8* locus. (A) Fosmids flanking the *Fgf8* locus mapped to the mm9 assembly of the mouse genome; (B) Examples of FISH images in DAPI-stained nuclei for the *Fgf8* -flanking probe pairs in stem zone, neural tube, and somite; (C) Box-plot of inter-probe distances (µm^2^) for *Fgf8* flanking probes in each tissue, indicating no difference in distances despite transcriptional down regulation of *Fgf8* in the neural tube and somites; (D) Boxplot showing that blocking FGFR signalling also did not alter inter-probe distances between fosmids flanking the *Fgf8* locus (images in Figure S5, and see text for discussion).
(TIF)

Figure S3 Expression patterns of *Fgf8* and its neighbouring genes *NPM3* and *Mgea5/OGA*. Localisation of mRNAs for *Fgf8*, *NPM3* and *Mgea5/OGA* in the caudal regions of the mouse embryos at (A, B, C) E8-8.25 and at E8.5 (A′, B′ C′). Asterisk indicates position of the node. All three genes are expressed in the stem zone at E8-8.25, at E8.5 *NPM3* and *Mgea5/OGA* transcripts are also detected more rostrally, but are still downregulated in the neural tube. (D) Table of primers used for *NPM3*, *Mgea5* and *Irx3* (Figure 7) in situ hybridisation.
(TIF)

Figure S4 Exposure to FGFR inhibitor PD173074 represses FGFR target *Spry2*, and does not alter chromatin compaction or nuclear position across the control *Hba-a1* locus. (A) Expression of *Spry2* in embryos following exposure to vehicle control DMSO or the FGFR antagonist PD173074. (B) Box-plot of inter-probe distances (µm^2^) for *Hba-a1* flanking probes in each tissue assessed in DMSO and PD173074 treated embryos, showing that FGFR signalling has no effect on chromatin compaction around the *Hba-a1* locus in the stem zone (p = 0.55) or neural tube (p = 0.08). (C) Graph of data distribution for fractional radius measurements in DMSO and PD173074 treated tissues, showing that the *Hba-a1* locus does not change nuclear position in the stem zone (p = 0.23) or neural tube (p = 0.83) after treatment with DMSO or FGFRI.
(TIF)

Figure S5 Comparison of *Fgf8* locus compaction and nuclear position in wildtype and in conditions lacking retinoid, FGFR, and both RA and FGFR signalling. Exemplar images of fosmid pairs across the *Fgf8* locus in nuclei from each tissue and condition assessed. Compaction of the genomic region around the *Fgf8* locus does not alter with *Fgf8* transcriptional activity, including in conditions in which retinoid or FGFR signalling or both are attenuated. However, nuclear position of the *Fgf8* locus is regulated by retinoid and FGF signalling (see text for details and data analysis in Figure 8). Scale bars = 2 microns in exemplar images and 1 micron in inset.
(TIF)

Figure S6 Detection of intronic *Fgf8* mRNA following inhibition of FGFR signalling. Intronic *Fgf8* mRNA was detected in the stem zone of (A) wildtype (n = 4/4) and (B) PD173074 treated embryos (n = 3/4). Scale bar = 100 microns.
(TIF)

Figure S7 *Pax6* and *Irx3* loci relocate to the centre of the nucleus in somite tissue treated with FGFR inhibitor. (A) Graph of data distribution for *Pax6* fractional radius measurements for somites in wild-type, *Raldh2−/*−, DMSO and PD173074 treated wild-type and PD173074 treated mutant tissues. These data show that FGF signalling is required for the proper localisation of the *Pax6* locus close to the nuclear periphery in somites. (B) Graph of data distribution for fractional radius measurements in DMSO and PD173074 treated tissues, showing that the *Irx3* locus displays a more central localisation within the nucleus when FGF signalling is blocked (p<0.05).
(TIF)

Figure S8 LaminB1 binding sites in the vicinity of *Pax6, Irx3, Fgf8* and control *Hba-a1* loci. Genomic regions around (A) *Pax6*, (B) *Irx3* (C) *Fgf8 and (D) Hba-a1* are not associated with Lamin B1 binding in ES cells or neural progenitors (analysis of data set from [29]).
(TIF)

Figure S9 PCR verification of fosmid sequences. Verification of *Fgf8, Irx3* and *Pax6* flanking fosmids was carried out using a standard PCR protocol (B) to amplify regions at the 5′ and 3′ends of fosmids obtained from the WIBR-1 Mouse Fosmid Library (Whitehead Institute/MIT Center for Genomic Research) (*Fgf8* C, *Irx3* D and *Pax6* E); Primer sequences and PCR product sizes are shown in A.
(TIF)

Table S1 Names and co-ordinates and sizes of fosmids used. All fosmid names are from Ensembl (r 45) http://jun2007.archive.ensembl.org/Mus_musculus/index.html). Fosmids highlighted with an asterisk were previously used in [13].
(DOC)

Table S2 Squared inter-probe distances of fosmids surrounding the *Pax6, Irx3, Hba-a1* and *Fgf8* loci in the stem zone, pre-neural tube (except *Hba-a1*), neural tube and somites. P-values are from Mann-Whitney analysis.
(DOC)

Table S3 Statistical significance between stem zone and neural tube in three embryos analysed for *Pax6, Irx3, Fgf8* and *Hba-a1* chromatin compaction.
(DOC)

Table S4 P-values from the paired-sample Wilcoxon signed-rank test showing statistical differences between individual embryo explant pairs analysed for *Pax6* chromatin compaction and nuclear localisation.
(DOC)
