# Correction: FGF Signalling Regulates Chromatin Organisation during Neural Differentiation via Mechanisms that Can Be Uncoupled from Transcription

**DOI:** 10.1371/annotation/c066bb84-13ea-4b36-a481-f149df8ce929

**Published:** 2013-08-12

**Authors:** Nishal S. Patel, Muriel Rhinn, Claudia I. Semprich, Pamela A. Halley, Pascal Dollé, Wendy A. Bickmore, Kate G. Storey

There were errors in the Funding section. The correct funding information is as follows: NSP is supported by a joint (to KGS & WAB) SULSA/BBSRC studentship at the University of Dundee; CIS is supported by a BBSRC DTA studentship; KGS is supported by MRC grants G0600234/1 and G1100552 and Wellcome Trust grant 083611/Z/07/Z. WAB is supported by a MRC unit programme and by ERC advanced grant 249956. PD and MR are supported by grants from the Agence Nationale de la Recherche and Fondation pour la Recherche Médicale. The funders had no role in study design, data collection and analysis, decision to publish, or preparation of the manuscript.

