# Supplementary figures and images for: FGF Signalling Regulates Chromatin Organisation during Neural Differentiation via Mechanisms that Can Be Uncoupled from Transcription
Source: PLoS Genet. 2013 Jul 18;9(7):e1003614. doi: 10.1371/journal.pgen.1003614 (PMC3715432; doi:10.1371/journal.pgen.1003614)

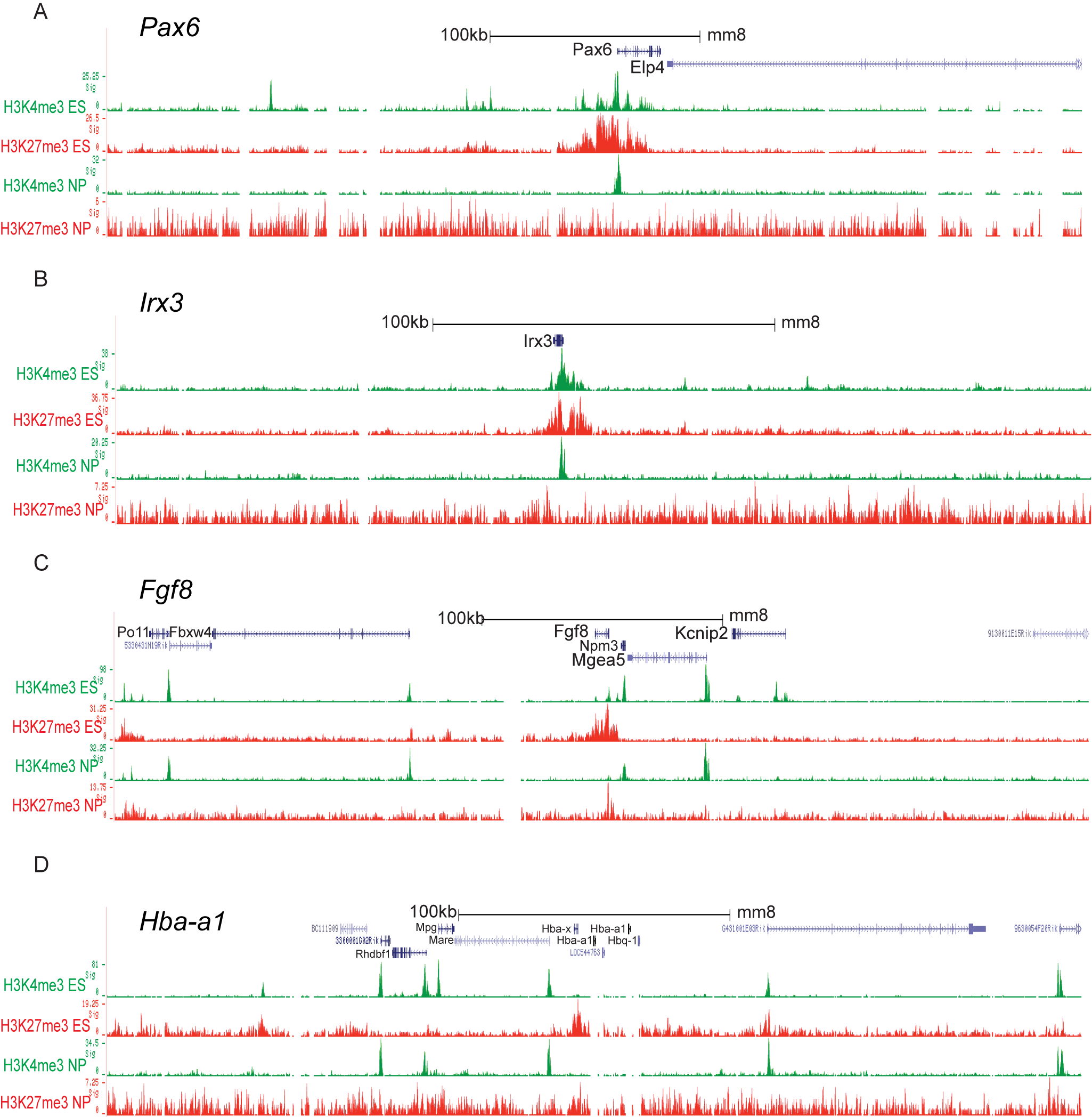

Supplement: Figure S1 — Histone modifications around key loci in ES cells and derived neural progenitors. Genomic co-ordinates are from the mm8 assembly of the mouse genome and histone modifications indicative of active H3K4me3 and silenced H3K27me3 regions around Pax6 (A), Irx3 (B), Fgf8 (C) and Hba-a1 (D) in mouse embryonic stem (ES) cells and ES cell derived neural progenitors (NP) (from dataset of [11]). (TIF) [file pgen.1003614.s001.tif]

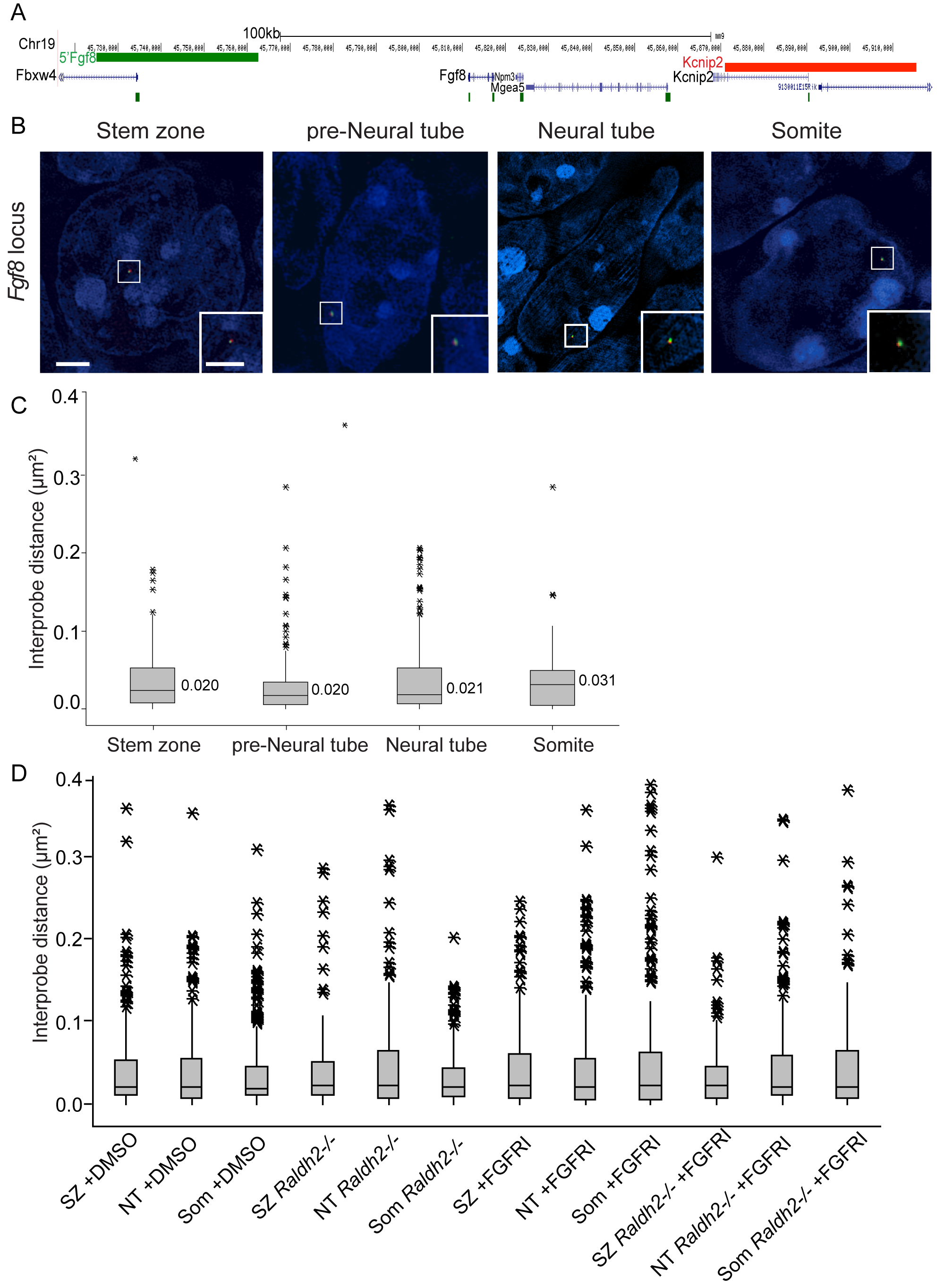

Supplement: Figure S2 — Assessment of chromatin compaction at the Fgf8 locus. (A) Fosmids flanking the Fgf8 locus mapped to the mm9 assembly of the mouse genome; (B) Examples of FISH images in DAPI-stained nuclei for the Fgf8 -flanking probe pairs in stem zone, neural tube, and somite; (C) Box-plot of inter-probe distances (µm2) for Fgf8 flanking probes in each tissue, indicating no difference in distances despite transcriptional down regulation of Fgf8 in the neural tube and somites; (D) Boxplot showing that blocking FGFR signalling also did not alter inter-probe distances between fosmids flanking the Fgf8 locus (images in Figure S5, and see text for discussion). (TIF) [file pgen.1003614.s002.tif]

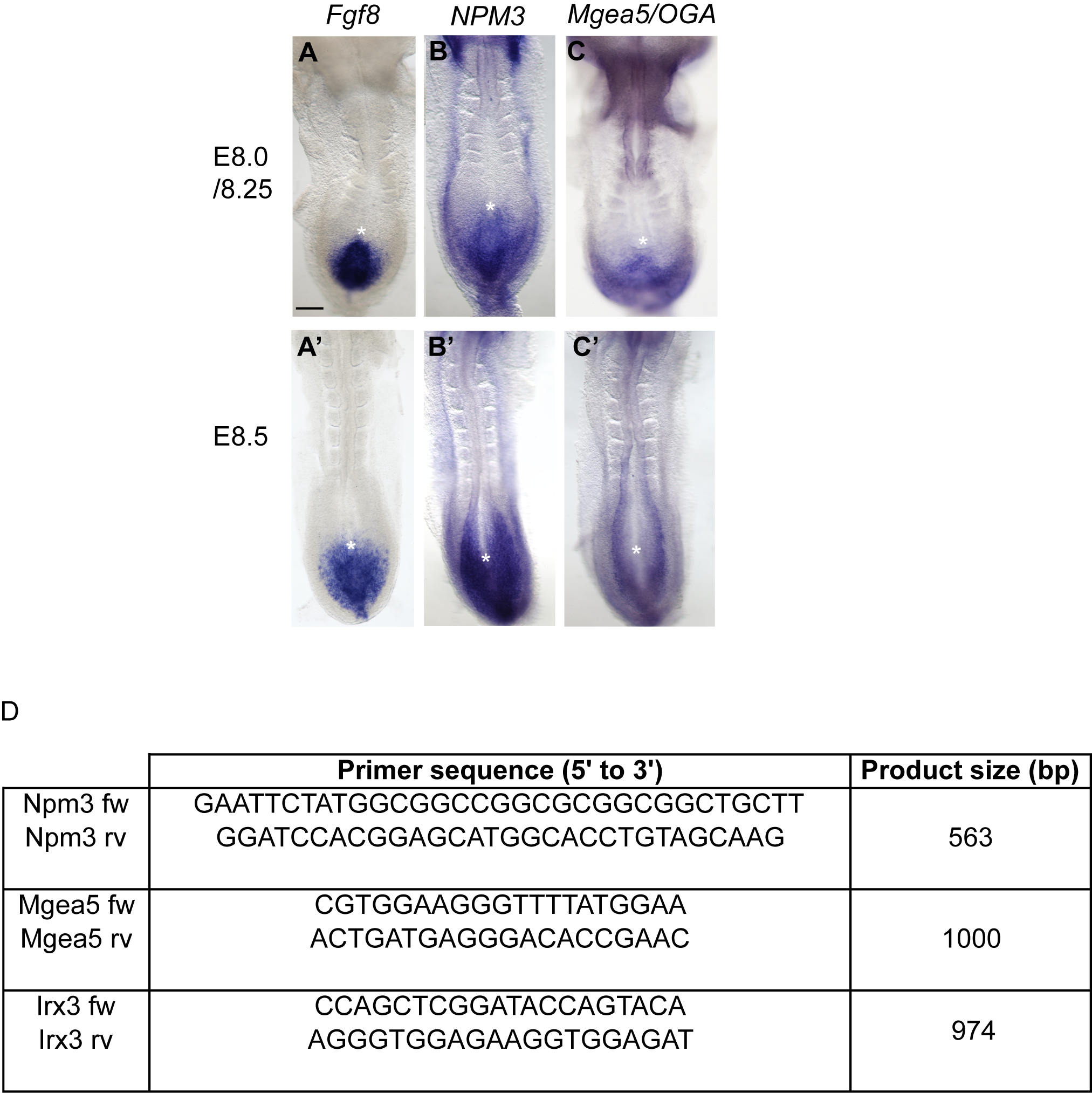

Supplement: Figure S3 — Expression patterns of Fgf8 and its neighbouring genes NPM3 and Mgea5/OGA. Localisation of mRNAs for Fgf8, NPM3 and Mgea5/OGA in the caudal regions of the mouse embryos at (A, B, C) E8-8.25 and at E8.5 (A′, B′ C′). Asterisk indicates position of the node. All three genes are expressed in the stem zone at E8-8.25, at E8.5 NPM3 and Mgea5/OGA transcripts are also detected more rostrally, but are still downregulated in the neural tube. (D) Table of primers used for NPM3, Mgea5 and Irx3 (Figure 7) in situ hybridisation. (TIF) [file pgen.1003614.s003.tif]

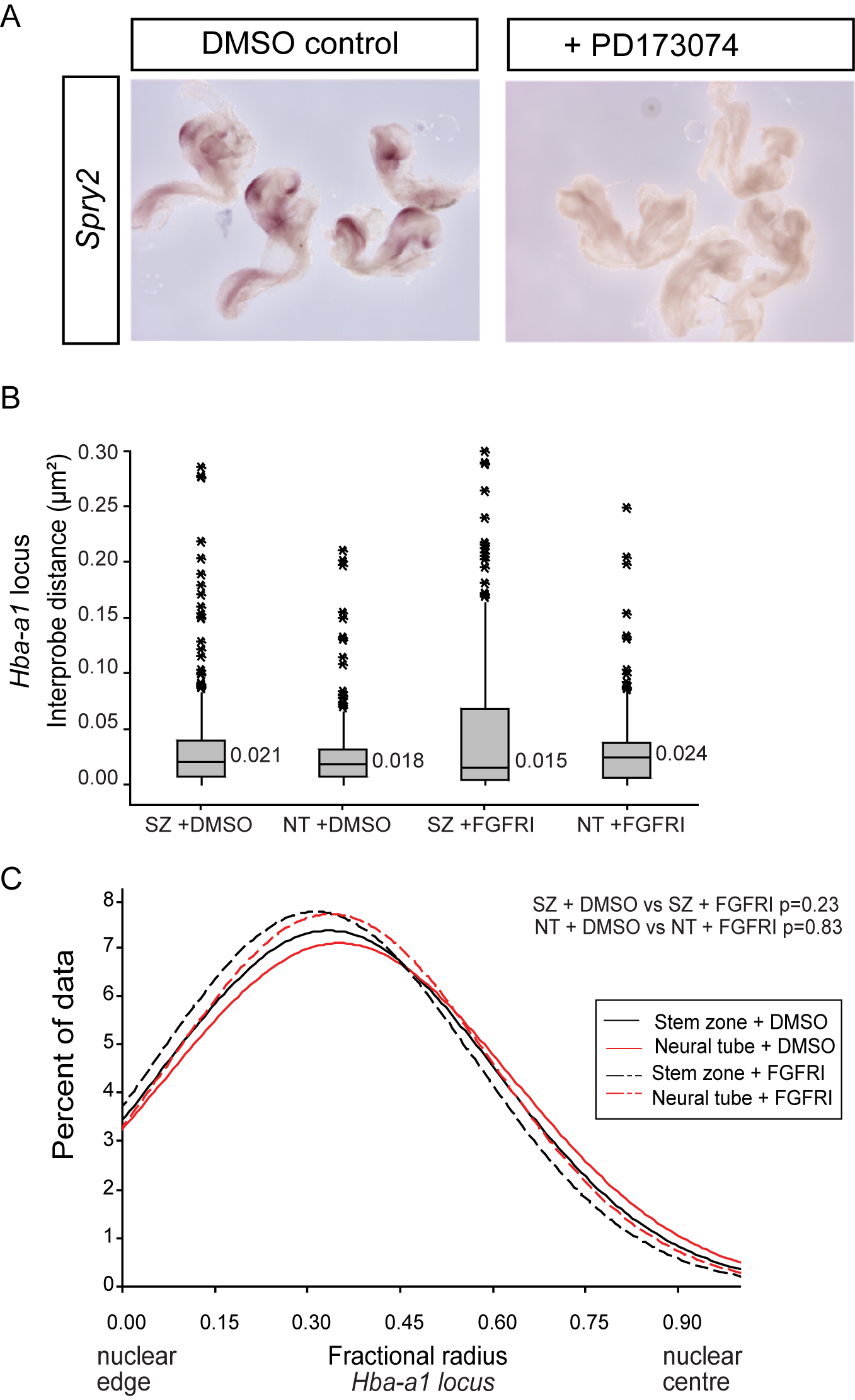

Supplement: Figure S4 — Exposure to FGFR inhibitor PD173074 represses FGFR target Spry2, and does not alter chromatin compaction or nuclear position across the control Hba-a1 locus. (A) Expression of Spry2 in embryos following exposure to vehicle control DMSO or the FGFR antagonist PD173074. (B) Box-plot of inter-probe distances (µm2) for Hba-a1 flanking probes in each tissue assessed in DMSO and PD173074 treated embryos, showing that FGFR signalling has no effect on chromatin compaction around the Hba-a1 locus in the stem zone (p = 0.55) or neural tube (p = 0.08). (C) Graph of data distribution for fractional radius measurements in DMSO and PD173074 treated tissues, showing that the Hba-a1 locus does not change nuclear position in the stem zone (p = 0.23) or neural tube (p = 0.83) after treatment with DMSO or FGFRI. (TIF) [file pgen.1003614.s004.tif]

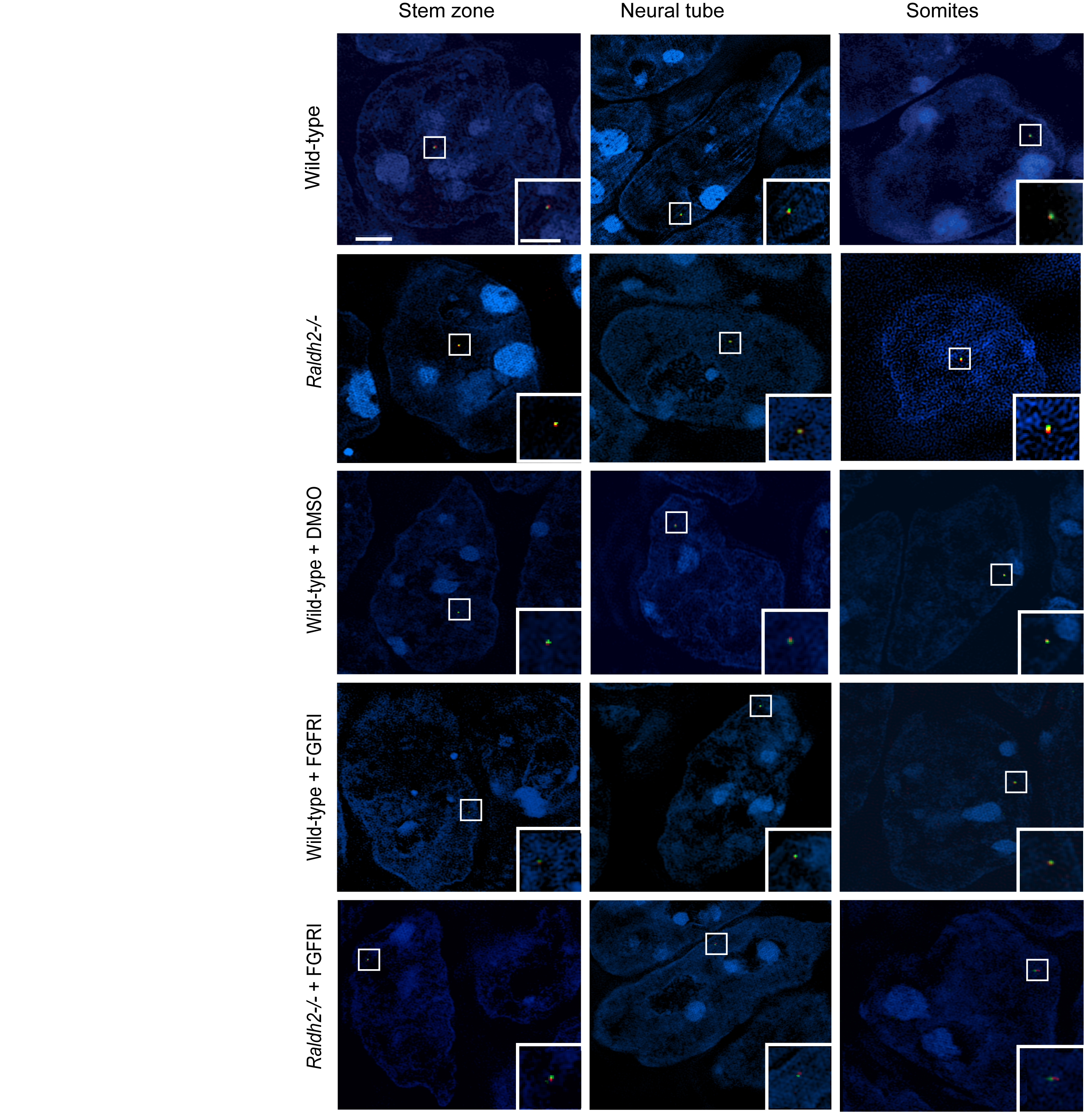

Supplement: Figure S5 — Comparison of Fgf8 locus compaction and nuclear position in wildtype and in conditions lacking retinoid, FGFR, and both RA and FGFR signalling. Exemplar images of fosmid pairs across the Fgf8 locus in nuclei from each tissue and condition assessed. Compaction of the genomic region around the Fgf8 locus does not alter with Fgf8 transcriptional activity, including in conditions in which retinoid or FGFR signalling or both are attenuated. However, nuclear position of the Fgf8 locus is regulated by retinoid and FGF signalling (see text for details and data analysis in Figure 8). Scale bars = 2 microns in exemplar images and 1 micron in inset. (TIF) [file pgen.1003614.s005.tif]

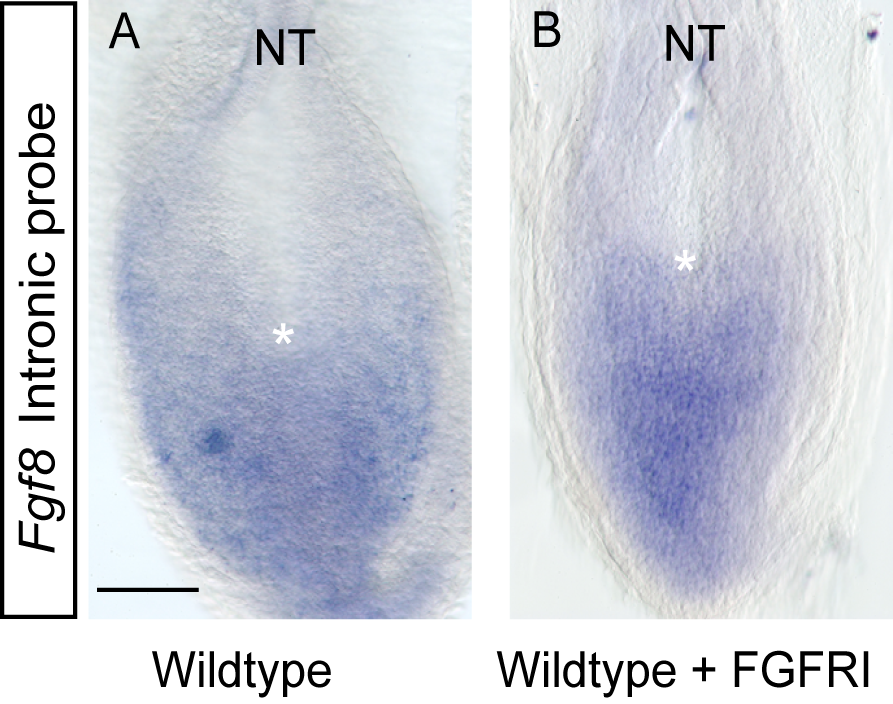

Supplement: Figure S6 — Detection of intronic Fgf8 mRNA following inhibition of FGFR signalling. Intronic Fgf8 mRNA was detected in the stem zone of (A) wildtype (n = 4/4) and (B) PD173074 treated embryos (n = 3/4). Scale bar = 100 microns. (TIF) [file pgen.1003614.s006.tif]

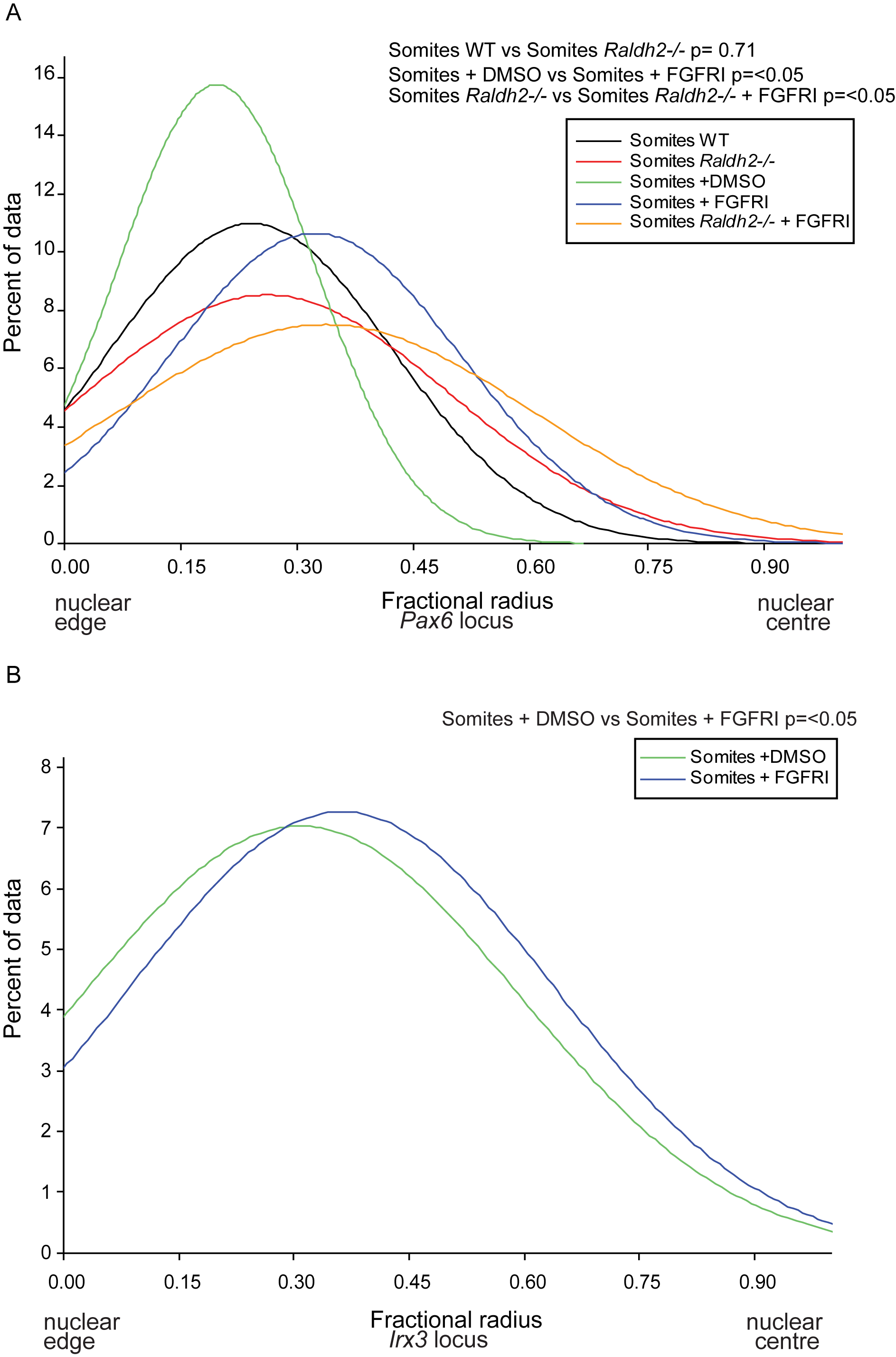

Supplement: Figure S7 — Pax6 and Irx3 loci relocate to the centre of the nucleus in somite tissue treated with FGFR inhibitor. (A) Graph of data distribution for Pax6 fractional radius measurements for somites in wild-type, Raldh2−/−, DMSO and PD173074 treated wild-type and PD173074 treated mutant tissues. These data show that FGF signalling is required for the proper localisation of the Pax6 locus close to the nuclear periphery in somites. (B) Graph of data distribution for fractional radius measurements in DMSO and PD173074 treated tissues, showing that the Irx3 locus displays a more central localisation within the nucleus when FGF signalling is blocked (p<0.05). (TIF) [file pgen.1003614.s007.tif]

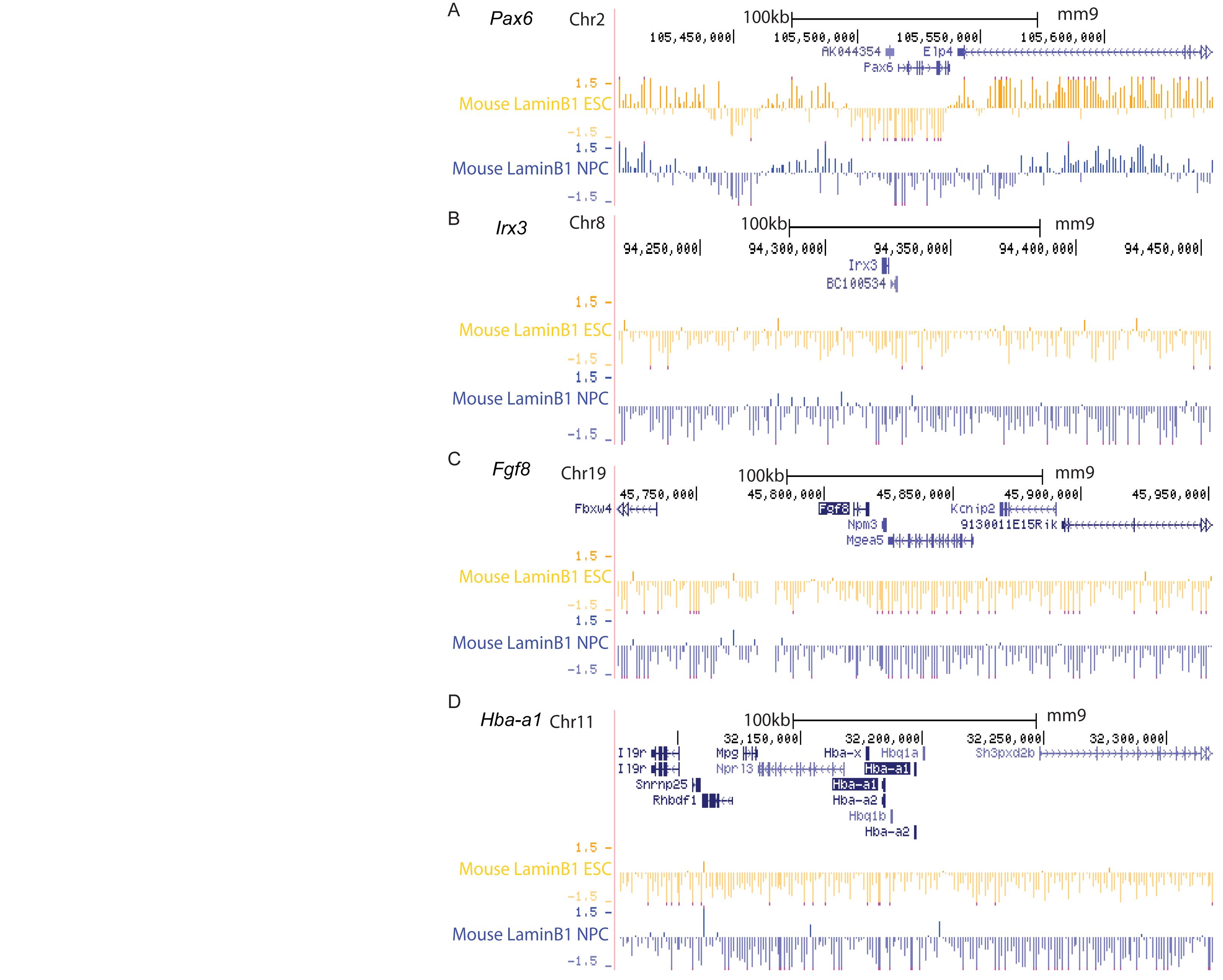

Supplement: Figure S8 — LaminB1 binding sites in the vicinity of Pax6, Irx3, Fgf8 and control Hba-a1 loci. Genomic regions around (A) Pax6, (B) Irx3 (C) Fgf8 and (D) Hba-a1 are not associated with Lamin B1 binding in ES cells or neural progenitors (analysis of data set from [29]). (TIF) [file pgen.1003614.s008.tif]

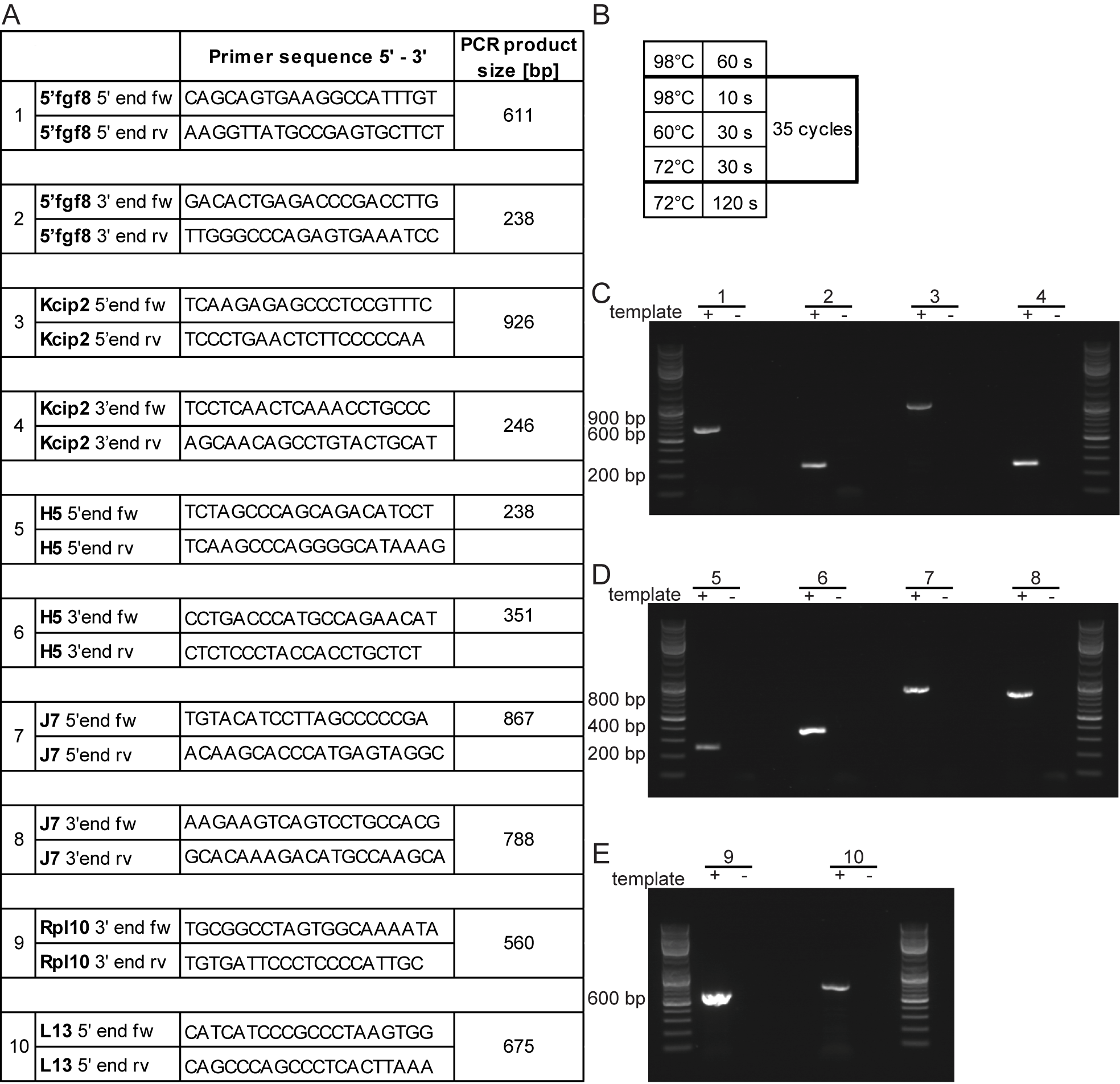

Supplement: Figure S9 — PCR verification of fosmid sequences. Verification of Fgf8, Irx3 and Pax6 flanking fosmids was carried out using a standard PCR protocol (B) to amplify regions at the 5′ and 3′ends of fosmids obtained from the WIBR-1 Mouse Fosmid Library (Whitehead Institute/MIT Center for Genomic Research) (Fgf8 C, Irx3 D and Pax6 E); Primer sequences and PCR product sizes are shown in A. (TIF) [file pgen.1003614.s009.tif]
