# Supplementary material for: FGF Signalling Regulates Chromatin Organisation during Neural Differentiation via Mechanisms that Can Be Uncoupled from Transcription
Source: PLoS Genet. 2013 Jul 18;9(7):e1003614. doi: 10.1371/journal.pgen.1003614 (PMC3715432; doi:10.1371/journal.pgen.1003614)
Supplement: Table S1 — Names and co-ordinates and sizes of fosmids used. All fosmid names are from Ensembl (r 45) http://jun2007.archive.ensembl.org/Mus_musculus/index.html). Fosmids highlighted with an asterisk were previously used in [13]. (DOC) [file pgen.1003614.s010.doc]

**Table S1**

|  | **Region** | **Whitehead Name** | **Ensembl Name** | **Co-ordinates** | | **Size (kb)** |
| --- | --- | --- | --- | --- | --- | --- |
|  |  |  |  | Start | Finish |  |
| Pax6 | Rpl10 | WI1-1659I15 | G135P603318C6 | 105415716 | 105453975 | 38259 |
|  | Elp4 | WI1-1248L13 | G135P603318C6 | 105519566 | 105562150 | 42584 |
| Fgf8 | 3' of *fgf8* | WI1-1134K6 | G13JP601465C10 | 45879774 | 45922841 | 43067 |
|  | Kcnip2 | WI1-2338G4 | G135600813E11 | 45725008 | 45762682 | 37674 |
| Alpha-globin | Hbq1 * | WI1-2903N21 | G135P603718B2 | 32196269 | 32241313 | 45044 |
|  | I19r * | WI1-2837A17 | G135P60495H4 | 32056569 | 32100774 | 44205 |
| Irx3 | H5 | WI1-1023H05 | G135P600146E10 | 94274005 | 94314898 | 40893 |
|  | J7 | WI1-0979J07 | G135P600826D9 | 94369661 | 94405340 | 35679 |
