# Supplementary material for: FGF Signalling Regulates Chromatin Organisation during Neural Differentiation via Mechanisms that Can Be Uncoupled from Transcription
Source: PLoS Genet. 2013 Jul 18;9(7):e1003614. doi: 10.1371/journal.pgen.1003614 (PMC3715432; doi:10.1371/journal.pgen.1003614)
Supplement: Table S2 — Squared inter-probe distances of fosmids surrounding the Pax6, Irx3, Hba-a1 and Fgf8 loci in the stem zone, pre-neural tube (except Hba-a1), neural tube and somites. P-values are from Mann-Whitney analysis. (DOC) [file pgen.1003614.s011.doc]

**Table S2**

| **Squared interprobe distances (d^2^)** | ***Pax6*** | ***Irx3*** | ***Hba-a1*** | ***Fgf8*** |
| --- | --- | --- | --- | --- |
| Stem zone | 0.036 |  |  | 0.020 |
| pre-Neural tube | 0.029 |  |  | 0.020 |
|  | **p=0.08** |  |  | **p=0.07** |
| pre-Neural tube | 0.029 |  |  | 0.020 |
| Neural tube | 0.071 |  |  | 0.021 |
|  | **p<0.01** |  |  | **p=0.14** |
| Neural tube | 0.071 | 0.096 | 0.021 | 0.021 |
| Somite | 0.032 | 0.041 | 0.022 | 0.031 |
|  | **p<0.01** | **p<0.01** | **p=0.14** | **p=0.27** |
| Stem zone | 0.036 | 0.029 | 0.018 | 0.020 |
| Neural tube | 0.071 | 0.096 | 0.021 | 0.021 |
|  | **p<0.01** | **P<0.01** | **P=0.85** | **p=0.85** |
