# Supplementary material for: FGF Signalling Regulates Chromatin Organisation during Neural Differentiation via Mechanisms that Can Be Uncoupled from Transcription
Source: PLoS Genet. 2013 Jul 18;9(7):e1003614. doi: 10.1371/journal.pgen.1003614 (PMC3715432; doi:10.1371/journal.pgen.1003614)
Supplement: Table S3 — Statistical significance between stem zone and neural tube in three embryos analysed for Pax6, Irx3, Fgf8 and Hba-a1 chromatin compaction. (DOC) [file pgen.1003614.s012.doc]

**Table S3**

|  | ***Pax6*** | ***Irx3*** | ***Fgf8*** | ***Hba-a1*** |
| --- | --- | --- | --- | --- |
| Stem zone vs neural tube embryo 1 | p<0.01 | p<0.01 | p=0.2 | p=0.84 |
| Stem zone vs neural tube embryo 2 | p<0.01 | p<0.01 | p=0.98 | p=0.61 |
| Stem zone vs neural tube embryo 3 | p<0.01 | p<0.01 | p=0.3 | p=0.12 |
