# Supplementary material for: FGF Signalling Regulates Chromatin Organisation during Neural Differentiation via Mechanisms that Can Be Uncoupled from Transcription
Source: PLoS Genet. 2013 Jul 18;9(7):e1003614. doi: 10.1371/journal.pgen.1003614 (PMC3715432; doi:10.1371/journal.pgen.1003614)
Supplement: Table S4 — P-values from the paired-sample Wilcoxon signed-rank test showing statistical differences between individual embryo explant pairs analysed for Pax6 chromatin compaction and nuclear localisation. (DOC) [file pgen.1003614.s013.doc]

**Table S4**

| **Explant pair** | **P value (compaction)** | **P value (localisation)** |
| --- | --- | --- |
| DMSO 1 VS RA 1 | p<0.001 | p<0.001 |
| DMSO 2 VS RA 2 | p<0.001 | p<0.001 |
| DMSO 3 VS RA 3 | p<0.001 | p<0.001 |
| DMSO 4 VS RA 4 | p<0.001 | p<0.001 |
| DMSO 5 VS RA 5 | p<0.001 | p<0.001 |
